# Supplementary material for: Cas9/gRNA-mediated genome editing of yeast mitochondria and Chlamydomonas chloroplasts
Source: PeerJ. 2020 Jan 6;8:e8362. doi: 10.7717/peerj.8362 (PMC6951285; doi:10.7717/peerj.8362)
Supplement: Supplemental Information 1 — The PCR fragments obtained from the integrated donor DNA were isolated from two replicated experiments and sequenced (#6 and #10 of Fig. 5B). The obtained sequences were aligned using Snapgene program (see text). Confirmed sequences of each of replicated experiments were identical to what was deduced from the expected integration of donor DNA through homologous recombination. [file peerj-08-8362-s001.pdf]

```
Start                SspI                                                                              SwaI
                                                                                                       DraI
5' NNNNNNNNNNNNCTNCNN NTTCTTTATTAATATTATTTGGTTGAATATAATTTCTATTTGATCTTGGATAAA TATCNTATAAA TTACCTTTATATATATTTATTATAATTATATTATTATTATTTAAATATGTTTTTATT
3' NNNNNNNNNNNNGANGNN NAAGAAATAATTATAATAAACCAACTTATATTAAAGATAAACTAGAACCTATTT ATAGNATATTT AATGGAAATATATATAAATAATATTAATATAATAATAATAAATTTATACAAAAATAA

   NNNNNNNNNNNNCTNCNN NTTCTTTATTAATATTATTTGGTTGAATATAATTTCTATTTGATCTTGGATAAA TATCNTATAAA TTACCTTTATATATATTTATTATAATTATATTATTATTATTTAAATATGTTTTTATTATATATTATTATTATTTATTTTTT GATAATTTCTGAT
1  ←                                                                                                                                                        gttttattatattattattatttattttt gataatttctgat
2  →             CTACNAANTTCTTTATTAATATTATTTGGTTGAATATAATTTCTATTTGATCTTGGATAAA TANCATATNAA TTACCTTTATATATATTTATTATAATTATATTATTATTATTTAAATATGTTTTTATTATATATTATTATTATTTATTTTTT GATAATTTCTGAT
6  ← TTATAACCATATACTACTA ATTCTTTATTAATATTATTTGGTTGAATATAATTTCTATTTGATCTTGGATAAA TATCATATAAA TTACCTTTATATATATTTATTATAATTATATTATTATTATTTAAATATGTTTTTATTATATATTATTATTATTTATTTTTT GATAATTTCTGAT
10 ← TTATAACCATATACTACTA ATTCTTTATTAATATTATTTGGTTGAATATAATTTCTATTTGATCTTGGATAAA TATCATATAAA TTACCTTTATATATATTTATTATAATTATATTATTATTATTTAAATATGTTTTTATTATATATTATTATTATTTATTTTTTTGATAATTTCTGAT
11 ←                 AA NTTCTTTATTAATATNAGTNGGTGGAATATAGTNTCTANN-GNT_NTNGATAAAATATCATATAAANTTACCTTTATAGATATTTATTATAATTATANNATTATTATTNAAANAGGTTTTTATTATANTAATATTATG-AATTTTT GATAATTTCTGAT
12 ← TTATAACCATATACTACTA ATTCTTTATTAATATTATTTGGTTGAATATAATTTCTATTTGATCTTGGATAAA TATCATATAAA TTACCTTTATATATATTTATTATAATTATATTATTATTATTTAAATATGTTTTTATTATATATTATTATTATTTATTTTTT GATAATTTCTGAT

                                                     BspHI                                                    PvuII
                                                                                                              AlwNI
                                                                                                              MspA1I             EcoP15I          BstYI
CAACTTTTT ATTTTTATTTTTATTTTT ATCC TTGCTAAAGGGATTTATTAATCATGAGAAAATTTTAATTCCTGTTGTTATTTATATAATTCATCCATACCATGTGTGATACCAGCAGCTGTTACAAATTC TAATAATAC
GTTGAAAAA TAAAAATAAAAATAAAAA TAGG AACGATTTCCCTAAATAATTAGTACTCTTTTAAAATTAAGGACAACAATAAATATATTAAGTAGGTATGGTACACACTATGGTCGTCGACAATGTTTAAG ATTATTATG

   CAACTTTTT ATTTTTATTTTTATTTTT ATCC TTGCTAAAGGGATTTATTAATCATGAGAAAATTTTAATTCCTGTTGTTATTTATATAATTCATCCATACCATGTGTGATACCAGCAGCTGTTACAAATTC TAATAATACCATATGATCTCTTTTTTCATTAGGATCTTTGATAA
1  ←caactttt atttttatttttattttt atcc ttgctaaagggatttattaatcatgagaaaattttaattcctgttgttatttatataattcatccataccatgtgtgataccagcagctgttacaaattc taataataccatatgatctctttttttcattaggatctttgataa
2  →CAACTTTTT ATTTTTATTTTTATTTTT ATCC TTGCTAAAGGGATTTATTAATCATGAGAAAATTTTAATTCCTGTTGTTATTTATATAATTCATCCATACCATGTGTGATACCAGCAGCTGTTACAAATTC TAATAATACCATATGATCTCTTTTTTCATTAGGATCTTTGATAA
6  ←CAACTTTTT ATTTTTATTTTTATTTTT ATCC TTGCTAAAGGGATTTATTAATCATGAGAAAATTTTAATTCCTGTTGTTATTTATATAATTCATCCATACCATGTGTGATACCAGCAGCTGTTACAAATTC TAATAATACCATATGATCTCTTTTTTCATTAGGATCTTTGATAA
10 ←CAACTTTTT ATTTTTATTTTTATTTTT ATCC TTGCTAAAGGGATTTATTAATCATGAGAAAATTTTAATTCCTGTTGTTATTTATATAATTCATCCATACCATGTGTGATNNCANCAGCTGTTACAAATTC TAATAATACCATATGATCTCTTTTTTCATTAGGATCTTTGATAA
11 ←CAACTTTTTTATTTNTATTTTTATTTTTTATCCCTTGCTAAAGGGATTTATTATTCATGAGGAAATTTTAATTCCTGTTGTTATTTATGTAATTCATCCATACCATGTGNGNTACCAGCAGCTGTTACAAATTC-TAATAATACCATATGATCTCTTTTTTCATTAGGATCTTTGATAA
12 ←CAACTTTTT ATTTTTATTTTTATTTTT ATCC TTGGTAAAGGGATTTATTAATCATGAGAAAATTTTAATTCCTGTTGTTATTTATATAATTCATCCATACCATGTGTGATGCCAGCAGCNGTTACAAATTT TAATAATACCATATGATCTCTTTTTTCATTAGGATCTTNTGATAA

              AflII
              SmlI                                                                        BbsI
AGCTGATTGTGTACTTAAGTAATGATTATC AGGTAATAATACAGGACCATCACCGATAGGTGTATTTTGTTGATAATGATCTGCTAATTGTACTGAACCGTCTTCGATATTATGTCTGATTTTGAAATTTACTTTGATACCAT
TCGACTAACACATGAATTCATTACTAATAG TCCATTATTATGTCCTGGTAGTGGCTATCCACATAAAACAACTATTACTAGACGATTAACATGACTTGGCAGAAGCTATAATACAGACTAAAACTTTAAATGAAACTATGGTA

   AGCTGATTGTGTACTTAAGTAATGATTATC AGGTAATAATACAGGACCATCACCGATAGGTGTATTTTGTTGATAATGATCTGCTAATTGTACTGAACCGTCTTCGATATTATGTCTGATTTTGAAATTTACTTTGATACCATTTTTTTGTTTATCAGCCATGATATATACATTATGTG
1  ←agctgattgtgtacttaagtaatgattatc aggtaataatacaggaccatcaccgataggtgtattttgttgataatgatctgctaattgtactgaaccgtcttcgatattatgtctgattttgaaatttactttgataccatttttttgtttatcagccatgatatatacattatgtg
2  →AGCTGATTGTGTACTTAAGTAATGATTATC AGGTAATAATACAGGACCATCACCGATAGGTGTATTTTGTTGATAATGATCTGCTAATTGTACTGAACCGTCTTCGATATTATGTCTGATTTTGAAATTTACTTTGATACCATTTTTTTGTTTATCAGCCATGATATATACATTATGTG
4  →                                                                                                                                             AATTTACTTTGATACCATTTTTTTGTTTATCAGCCATGATATATACATTATGTG
6  ←AGCTGATTGTGTACTTAAGTAATGATTATC AGGTAATAATACAGGACCNNCANNG
7  →                                                                                                                                             AATTTACTTTGATACCATTTTTTTGTTTATCAGCCATGATATATACATTATGNG
8  ←                                                                                      NNGTACTGAACCGTCTTCGATATTATGTCTGATTTTGAAATTTACTTTGATACCATTTTTTTGTTTATCAGCCATGATATATACATTATGTG
9  ←                                                                                        GTACTGAACCGTCTTCGATATTATGTCTGATTTTGAAATTTACTTTGATACCATTTTTTTGTTTATCAGCCATGATATATACATTATGTG
10 ←AGCTGATTGTGTACTTAAGTAATGATTATC AGGTAATAATACAGGACCATCACCGATAGGTGTATTTTGTTGATAATGATCTGCTAATTGTACTGAACCGTCTTCGATATTATGTCTGATTTTGAAATTTACTTTGATACCATTTTTTTGTTTATCAGCCATGATATATACATTATGTG
11 ←AGGTGATTGTGTACGTAAGTAATGATTATC AGGTAATAATACAGGACCNNCACCGATAGGTGTATTTTGTTGATAATGATCTGNTAATTGTACTGAACCGTCTTCGATATTATGTCTGATTTTGAAATTTACTTTGATACCATTTTTTTGTTTATCAGCCATGATATATGCATTATGTG
12 ←AGCTGATTGTGTACTTAAGTAANGANTATNNAGGNAATAANNCNGGNNCNNCAC

                                                 ClaI
                                                 BspDI                                  BstBI                                                                          BsaAI
                                                                                                                                                                       SnaBI       BsrDI
AATTATAATTATATTCTAATTTATGACCTAAGATATTACCATCTTCTTTGAAATCGATACCTTTTAACTCGATTCTATTTACTAATGTATCACCTTCGAATTTTACTTCAGCTCTTGTTTTATAATTACCATCATCTTTGAAGA
TTAATATTAATATAAGATTAAATACTGGATTCTATAATGGTAGAAGAAACTTTAGCTATGGAAAATTGAGCTAAGATAAATGATTACATAGTGGAAGCTTAAAATGAAGTCGAGAACAAAATATTAATGGTAGTAGAAACTTCT

   AATTATAATTATATTCTAATTTATGACCTAAGATATTACCATCTTCTTTGAAATCGATACCTTTTAACTCGATTCTATTTACTAATGTATCACCTTCGAATTTTACTTCAGCTCTTGTTTTATAATTACCATCATCTTTGAAGAAGATTGTTCTTTCTTGTACGTAACCTTCAGGCATTG
1  ←aattataattatattctaatttatgacctaagatattaccatcttctttgaaatcgataccttttaactcgattctatttactaatgtatcaccttcgaattttacttcagctcttgttttataattaccatcatctttgaagaagattgttctttcttgtacgtaaccttcaggcattg
2  →AATTATAATTATATTCTAATTTATGACCTAAGATATTACCATCTTCTTTGAAATCGATACCTTTTAACTCGATTCTATTTACTAATGTATCACCTTCGAATTTTACTTCAGCTCTTGTTTTATAATTACCATCATCTTTGAAGAAGATTGTTCTTTCTTGTACGTAACCTTCAGGCATTG
4  →AATTATAATTATATTCTAATTTATGACCTAAGATATTACCATCTTCTTTGAAATCGATACCTTTTAACTCGATTCTATTTACTAATGTATCACCTTCGAATTTTACTTCAGCTCTTGTTTTATAATTACCATCATCTTTGAAGAAGATTGTTCTTTCTTGTACGTAACCTTCAGGCATTG
7  →AATTATAATTATATTCTAATTTATGACCTAAGATATTACCATCTTCTTTGAAATCGATACCTTTTAACTCGATTCTATTTACTAATGTATCACCTTCGAATTTTACTTCAGCTCTTGTTTTATAATTACCATCATCTTTGAAGAAGATTGTTCTTTCTTGTACGTAACCTTCAGGCATTG
8  ←AATTATAATTATATTCTAATTTATGACCTAAGATATTACCATCTTCTTTGAAATCGATACCTTTTAACTCGATTCTATTTACTAATGTATCACCTTCGAATTTTACTTCAGCTCTTGTTTTATAATTACCATCATCTTTGAAGAAGATTGTTCTTTCTTGTACGTAACCTTCAGGCATTG
9  ←AATTATAATTATATTCTAATTTATGACCTAAGATATTACCATCTTCTTTGAAATCGATACCTTTTAACTCGATTCTATTTACTAATGTATCACCTTCGAATTTTACTTCAGCTCTTGTTTTATAATTACCATCATCTTTGAAGAAGATTGTTCTTTCTTGTACGTAACCTTCAGGCATTG
10 ←AATTATAATTATATTCTAATTTATGACCTAAGATATTACCATCTTCTTTGAAATCGATACCTTTTAACTCGATTCTATTTACTAATGTATCACCTTCGAATTTTACTTCAGCTCTTGTTTTATAATTACCATCATCTTTGAAGAAGATTGTTCTTTCTTGTACGTAACCTTCAGGCATTG
11 ←AATTATAATTATATTCTAATTTATGACCTAAGATATTACCATCTTCTTTGAAATCGATACCTTTTAACTCGATTCTATTTACTAATGTATCACCTTCGAATTTTACTTCAGCTCTTGTTTTATAATTACCATCATCTTNGAAGAAGATTGTTCTTTCTTGTACGTAACCTTCAGGCATTG
```

```
                                                EcoRV                                                          BsrGI                                                        End
   CTGATTTGAAGAAATCATGTTGTTTCATATGATCAGGATATCTTGCGAAACATTGTACTCCATATCCGAATGTTGTTACTAATGTAGGTCAAGGTACAGGTAATTTACCTGTTGTACAGATGAATTTTAATGTTAATTTACCAT
   GACTAAACTTCTTTAGTACAACAAAGTATACTAGTCCTATAGAACGCTTTGTAACATGAGGTATAGGCTTACAACAATGATTACATCCAGTTCCATGTCCATTAAATGGACAACATGTCTACTTAAAATTACAATTAAATGGTA

   CTGATTTGAAGAAATCATGTTGTTTCATATGATCAGGATATCTTGCGAAACATTGTACTCCATATCCGAATGTTGTTACTAATGTAGGTCAAGGTACAGGTAATTTACCTGTTGTACAGATGAATTTTAATGTTAATTTACCATATGTAGCATCACCTTCNCCCNNNNNNNCTGA
1  ← ctgatttgaagaaatcatgttgtttcatatgatcaggatatcttgcgaaacattgtactccatatccgaatgttgttactaatgtaggtcaaggtacaggtaatttacctgttgtacagatgaattttaatgttaatttaccatatgtagcatcaccttcacc
2  → CTGATTTGAAGAAATCATGTTGTTTCATATGATCAGGATATCTTGCGAAACATTGTACTCCATATCCGAATGTTGTTACTAATGTANGTCAAGGTACAGGTAATTTACCTGTTGTACAGATGA-NTTTAATGTTAATTTACCATATGTAGCATCACCTTCACCCNNTCACC
3  →                                                                                                                                       GTTAATTTACCATATGTAGCATCACCTTCACC
4  → CTGATTTGAAGAAATCATGTTGTTTCATATGATCAGGATATCTTGCGAAACATTGTACTCCATATCCGAATGTTGTTACTAATGTAGGTCAAGGTACAGGTAATTTACCTGTTGTACAGATGAATTTTAATGTTAATTTACCATATGTAGCATCACCTTCACC
5  →                                                                                                                              ANTTTAATGNNNNNTTACCATATGTAGCATCACCTTCACC
7  → CTGATTTGAAGAAATCATGTTGTTTCATATGATCAGGATATCTTGCGAAACATTGTACTCCATATCCGAATGTTGTTACTAATGTAGGTCAAGGTACAGGTAATTTACCTGTTGTACAGATGAATTTTAATGTTAATTTACCATATGTAGCATCACCTTCACC
8  ← CTGATTTGAAGAAATCATGTTGTTTCATATGATCAGGATATCTTGCGAAACATTGTACTCCATATCCGAATGTTGTTACTAATGTAGGTCAAGGTACAGGTAATTTACCTGTTGTACAGATGAATTTTAATGTTAATTTACCATATGTAGCATCACCTTCACC
9  ← CTGATTTGAAGAAATCATGTTGTTTCATATGATCAGGATATCTTGCGAAACATTGTACTCCATATCCGAATGTTGTTACTAATGTAGGTCAAGGTACAGGTAATTTACCTGTTGTACAGATGAATTTTAATGTTAATTTACCATATGTAGCATCACCTTCACC
10 ← CTGATTTGAAGAAATCATGTTGTTTCATATGATCAGGATATCTTGCGAAACATTGTACTCCNNNNNCGAATGTTGTTACTAATGTAGGTCAAGGTACAGGTAATTTACCTGT-GTAC
11 ← CTGATTTGAAGAAATCATGTTGTNTCATATGATCAGGATATCTTGCGAAACAT-GTACTCCATNTNNGNANNGAGTTCCTNATGTAGGTCAAGGTACAGGNNA
```

Original Sequence: HS alignment.dna

1: Template DNA_COX1 GFPm ←
1093 bases
325 .. 1066 (1 mismatch)

2: 3-F →
886 bases
15 .. 883 (12 mismatches, 2 gaps)

3: 1-15 →
464 bases
24 .. 55 (1 mismatch)

4: 1-12 →
829 bases
22 .. 418 (1 mismatch)

5: 2-15 →
467 bases
20 .. 59 (7 mismatches)

6: 3-14 ←
453 bases
18 .. 424 (22 mismatches)

7: 2-12 →
831 bases
24 .. 420 (2 mismatches)

8: 1-C ←
834 bases
386 .. 820 (3 mismatches)

9: 2-C ←
832 bases
385 .. 817 (1 mismatch)

10: 4-11 ←
887 bases
30 .. 857 (26 mismatches, 2 gaps)

11: 3-11 ←
897 bases
47 .. 845 (47 mismatches, 10 gaps)

12: 4-14 ←
455 bases
23 .. 428 (34 mismatches, 1 gap)
