# Supplementary material for: Cas9/gRNA-mediated genome editing of yeast mitochondria and Chlamydomonas chloroplasts
Source: PeerJ. 2020 Jan 6;8:e8362. doi: 10.7717/peerj.8362 (PMC6951285; doi:10.7717/peerj.8362)
Supplement: Supplemental Information 2 [file peerj-08-8362-s002.pdf]

```
 SpCas9     (1) ATGGATAAGAAATACTCAATAGGCTTAGATATCGGCACAAATAGCGTCGGATGGGCGGTGATCACTGATGAATATAAGGTTCCGTCTAAAAAGTTCAAGG
  Cas9c     (1) ATGGACAAAAAATACTCAATTGGTTTAGATATTGGTACAAATTCAGTTGGTTGGGCTGTTATTACAGATGAATATAAAGTTCCAAGTAAAAAATTTAAAG

 SpCas9   (101) TTCTGGGAAATACAGACCGCCACAGTATCAAAAAAAATCTTATAGGGGCTCTTTTATTTGACAGTGGAGAGACAGCGGAAGCGACTCGTCTCAAACGGAC
  Cas9c   (101) TTTTAGGTAATACAGATCGTCACTCAATTAAGAAAAACTTAATTGGTGCTTTATTATTTGATTCAGGTGAAACAGCTGAAGCTACACGTTTAAAACGTAC

 SpCas9   (201) AGCTCGTAGAAGGTATACACGTCGGAAGAATCGTATTTGTTATCTACAGGAGATTTTTTCAAATGAGATGGCGAAAGTAGATGATAGTTTCTTTCATCGA
  Cas9c   (201) AGCTCGTCGTCGTTATACACGTCGTAAAAATCGTATTTGTTATTTACAAGAAATTTTCTCAAATGAAATGGCTAAAGTTGATGATTCATTTTTTCACCGT

 SpCas9   (301) CTTGAAGAGTCTTTTTTGGTGGAAGAAGACAAGAAGCATGAACGTCATCCTATTTTTGGAAATATAGTAGATGAAGTTGCTTATCATGAGAAATATCCAA
  Cas9c   (301) TTAGAAGAATCATTTTTAGTTGAAGAAGATAAAAAACACGAACGTCACCCAATTTTTGGTAATATTGTTGATGAAGTTGCTTATCACGAAAAATATCCAA

 SpCas9   (401) CTATCTATCATCTGCGAAAAAAATTGGTAGATTCTACTGATAAAGCGGATTTGCGCTTAATCTATTTGGCCTTAGCGCATATGATTAAGTTTCGTGGTCA
  Cas9c   (401) CAATTTATCACTTACGTAAAAAATTAGTTGATTCAACTGATAAAGCTGATTTACGTTTAATTTATTTAGCTTTAGCTCACATGATTAAATTCCGTGGTCA

 SpCas9   (501) TTTTTTGATTGAGGGAGATTTAAATCCTGATAATAGTGATGTGGACAAACTATTTATCCAGTTGGTACAAACCTACAATCAATTATTTGAAGAAAACCCT
  Cas9c   (501) CTTCTTAATTGAAGGTGATTTAAACCCAGATAATTCAGATGTTGACAAATTATTCATTCAATTAGTTCAAACATATAATCAATTATTTGAAGAAAATCCA

 SpCas9   (601) ATTAACGCAAGTGGAGTAGATGCTAAAGCGATTCTTTCTGCACGATTGAGTAAATCAAGACGATTAGAAAATCTCATTGCTCAGCTCCCCGGTGAGAAGA
  Cas9c   (601) ATTAATGCTTCAGGTGTTGATGCTAAAGCAATTTTATCAGCTCGTTTATCAAAATCACGTCGTTTAGAAAACTTAATTGCTCAATTACCAGGTGAAAAGA

 SpCas9   (701) AAAATGGCTTATTTGGGAATCTCATTGCTTTGTCATTGGGTTTGACCCCTAATTTTAAATCAAATTTTGATTTGGCAGAAGATGCTAAATTACAGCTTTC
  Cas9c   (701) AAAATGGTTTATTCGGTAACTTAATTGCATTATCATTAGGTTTAACACCAAATTTCAAATCAAACTTCGATTTAGCTGAAGATGCTAAATTACAATTATC

 SpCas9   (801) AAAAGATACTTACGATGATGATTTAGATAATTTATTGGCGCAAATTGGAGATCAATATGCTGATTTGTTTTTGGCAGCTAAGAATTTATCAGATGCTATT
  Cas9c   (801) AAAAGATACATACGATGATGATTTAGATAACTTATTAGCACAAATTGGTGATCAATATGCTGATTTATTCTTAGCTGCTAAAAACTTATCAGATGCTATT

 SpCas9   (901) TTACTTTCAGATATCCTAAGAGTAAATACTGAAATAACTAAGGCTCCCCTATCAGCTTCAATGATTAAACGCTACGATGAACATCATCAAGACTTGACTC
  Cas9c   (901) TTATTATCAGATATTTTACGTGTTAATACAGAAATTACAAAAGCTCCATTATCAGCTTCAATGATTAAACGTTATGATGAACACCACCAAGATTTAACAT

 SpCas9  (1001) TTTTAAAAGCTTTAGTTCGACAACAACTTCCAGAAAAGTATAAAGAAATCTTTTTTGATCAATCAAAAAACGGATATGCAGGTTATATTGATGGGGGAGC
  Cas9c  (1001) TATTAAAAGCTTTAGTTCGTCAACAATTACCTGAAAAATACAAAGAAATTTTCTTCGATCAATCTAAAAATGGTTATGCTGGTTATATTGATGGTGGTGC

 SpCas9  (1101) TAGCCAAGAAGAATTTTATAAATTTATCAAACCAATTTTAGAAAAAATGGATGGTACTGAGGAATTATTGGTGAAACTAAATCGTGAAGATTTGCTGCGC
  Cas9c  (1101) TTCACAAGAAGAATTCTATAAATTCATTAAACCTATTTTAGAAAAAATGGATGGTACAGAAGAATTATTAGTTAAATTAAATCGTGAAGATTTATTACGT

 SpCas9  (1201) AAGCAACGGACCTTTGACAACGGCTCTATTCCCCATCAAATTCACTTGGGTGAGCTGCATGCTATTTTGAGAAGACAAGAAGACTTTTATCCATTTTTAA
  Cas9c  (1201) AAACAACGTACATTTGATAATGGTTCAATTCCTCACCAAATTCATTTAGGTGAATTACACGCAATTTTACGTCGTCAAGAAGATTTTTATCCATTCTTAA

 SpCas9  (1301) AAGACAATCGTGAGAAGATTGAAAAAATCTTGACTTTTCGAATTCCTTATTATGTTGGTCCATTGGCGCGTGGCAATAGTCGTTTTGCATGGATGACTCG
  Cas9c  (1301) AAGATAATCGTGAAAAAATTGAAAAAATTTTAACATTTCGTATTCCATATTATGTAGGTCCATTAGCTCGTGGTAATTCACGTTTCGCTTGGATGACACG

 SpCas9  (1401) GAAGTCTGAAGAAACAATTACCCCATGGAATTTTGAAGAAGTTGTCGATAAAGGTGCTTCAGCTCAATCATTTATTGAACGCATGACAAACTTTGATAAA
  Cas9c  (1401) TAAATCTGAAGAAACAATTACACCTTGGAATTTTGAAGAAGTTGTTGATAAAGGTGCTAGTGCTCAATCATTTATTGAACGTATGACAAATTTCGACAAA

 SpCas9  (1501) AATCTTCCAAATGAAAAAGTACTACCAAAACATAGTTTGCTTTATGAGTATTTTACGGTTTATAACGAATTGACAAAGGTCAAATATGTTACTGAAGGAA
  Cas9c  (1501) AACTTACCAAATGAAAAAGTTTACCAAAACACTCATTATTATATGAATATTTCACAGTTTATAATGAATTAACAAAAGTTAAATATGTTACAGAAGGTA

 SpCas9  (1601) TGCGAAAACCAGCATTTCTTTCAGGTGAACAGAAGAAAGCCATTGTTGATTTACTCTTCAAAACAAATCGAAAAGTAACCGTTAAGCAATTAAAAGAAGA
  Cas9c  (1601) TGCGTAAACCTGCATTTTTAAGTGGTGAACAAAAGAAAGCTATTGTTGACTTATTATTCAAAACAAATCGTAAAGTTACAGTTAAACAATTAAAAGAAGA

 SpCas9  (1701) TTATTTCAAAAAAATAGAATGTTTTGATAGTGTTGAAATTTCAGGAGTTGAAGATAGATTTAATGCTTCATTAGGTACCTACCATGATTTGCTAAAAATT
  Cas9c  (1701) TTACTTTAAGAAAATTGAATGTTTTGATTCAGTAGAAATTTCAGGTGTAGAAGATCGTTTCAATGCTTCATTAGGTACATACCACGATTTATTAAAAATT

 SpCas9  (1801) ATTAAAGATAAAGATTTTTTGGATAATGAAGAAAATGAAGATATCTTAGAGGATATTGTTTTAACATTGACCTTATTTGAAGATAGGGAGATGATTGAGG
  Cas9c  (1801) ATTAAAGACAAAGACTTTTTAGATAATGAAGAAAATGAAGATATTTTAGAAGATATTGTTTTAACATTAACATTATTCGAAGATCGTGAAATGATTGAAG

 SpCas9  (1901) AAAGACTTAAAACATATGCTCACCTCTTTGATGATAAGGTGATGAAACAGCTTAAACGTCGCCGTTATACTGGTTGGGGACGTTTGTCTCGAAAATTGAT
  Cas9c  (1901) AACGTTTAAAAACATATGCTCACTTATTTGATGATAAAGTTATGAAACAATTAAAACGTCGTCGTTACACAGGTTGGGGTCGTTTATCTCGTAAATTAAT

 SpCas9  (2001) TAATGGTATTAGGGATAAGCAATCTGGCAAAACAATATTAGATTTTTTGAAATCAGATGGTTTTGCCAATCGCAATTTTATGCAGCTGATCCATGATGAT
  Cas9c  (2001) TAACGGTATTCGTGACAAACAATCAGGTAAAACAATTTTAGATTTCTTAAAATCAGATGGTTTTGCTAATCGTAACTTTATGCAATTAATTCACGATGAT

 SpCas9  (2101) AGTTTGACATTTAAAGAAGACATTCAAAAAGCACAAGTGTCTGGACAAGGCGATAGTTTACATGAACATATTGCAAATTTAGCTGGTAGCCCTGCTATTA
  Cas9c  (2101) TCTTTAACATTCAAAGAAGATATTCAAAAAGCTCAAGTTTCAGGTCAAGGTGATTCATTACACGAACACATTGCTAACTTAGCTGGTTCTCCAGCTATTA

 SpCas9  (2201) AAAAAGGTATTTTACAGACTGTAAAAGTTGTTGATGAATTGGTCAAAGTAATGGGGCGGCATAAGCCAGAAAATATCGTTATTGAAATGGCACGTGAAAA
  Cas9c  (2201) AAAAAGGTATTTTACAAACAGTTAAAGTTGTAGATGAATTAGTAAAAGTAATGGGTCGTCACAAACCAGAAAACATTGTTATTGAAATGGCACGTGAAAA

 SpCas9  (2301) TCAGACAACTCAAAAGGGCCAGAAAAATTCGCGAGAGCGTATGAAACGAATCGAAGAAGGTATCAAAGAATTAGGAAGTCAGATTCTTAAAGAGCATCCT
  Cas9c  (2301) TCAAACAACACAAAAAGGTCAAAGAACTCACGTGAACGTATGAAACGTATTGAAGAAGGTATTAAAGAATTAGGTTCACAAATTTTAAAGAACACCCA

 SpCas9  (2401) GTTGAAAATACTCAATTGCAAAATGAAAAGCTCTATCTCTATTATCTCCAAAATGGAAGAGACATGTATGTGGACCAAGAATTAGATATTAATCGTTTAA
  Cas9c  (2401) GTTGAAAATACACAATTACAAAACGAAAAATTATATTTATACTATTTACAAAATGGTCGTGATATGTATGTAGATCAAGAATTAGATATTAACCGTTTAT

 SpCas9  (2501) GTGATTATGATGTCGATCACATTGTTCCACAAAGTTTCCTTAAAGACGATTCAATAGACAATAAGGTCTTAACGCGTTCTGATAAAAATCGTGGTAAATC
  Cas9c  (2501) CAGATTATGATGTTGATCACATTGTTCCACAATCTTTCTTAAAAGACGATTCAATTGATAACAAAGTTTTAACACGTTCAGATAAAAACCGTGGTAAATC

 SpCas9  (2601) GGATAACGTTCCAAGTGAAGAAGTAGTCAAAAAGATGAAAAACTATTGGAGACAACTTCTAAACGCCAAGTTAATCACTCAACGTAAGTTTGATAATTTA
  Cas9c  (2601) AGATAATGTACCATCAGAAGAAGTAGTTAAGAAAATGAAAAACTATTGGCGTCAATTATTAAATGCAAAATTAATTACACAACGTAAATTCGATAACTTA

 SpCas9  (2701) ACGAAAGCTGAACGTGGAGGTTTGAGTGAACTTGATAAAGCTGGTTTTATCAAACGCCAATTGGTTGAAACTCGCCAAATCACTAAGCATGTGGCACAAA
  Cas9c  (2701) ACAAAAGCTGAACGTGGTGGTTTATCAGAATTAGACAAAGCTGGTTTCATTAAACGTCAATTAGTAGAAACACGTCAAATTACTAAACACGTTGCTCAAA

 SpCas9  (2801) TTTTGGATAGTCGCATGAATACTAAATACGATGAAAATGATAAACTTATTCGAGAGGTTAAAGTGATTACCTTAAAATCTAAATTAGTTTCTGACTTCCG
  Cas9c  (2801) TTTTAGACTCTCGTATGAATACAAAATATGATGAAAATGATAAATTAATTCGTGAAGTTAAAGTTATTACATTAAAATCAAAATTAGTATCAGATTTCCG

 SpCas9  (2901) AAAAGATTTCCAATTCTATAAAGTACGTGAGATTAACAATTACCATCATGCCCATGATGCGTATCTAAATGCCGTCGTTGGAACTGCTTTGATTAAGAAA
  Cas9c  (2901) TAAAGATTTCCAATTCTACAAAGTTCGTGAAATTAACAACTATCACCACGCTCACGATGCTTACTTAAATGCTGTTGTTGGTACTGCATTAATTAAAAAA
```

```
SpCas9  (3001) TATCCAAAACTTGAATCGGAGTTTGTCTATGGTGATTATAAAGTTTATGATGTTCGTAAAATGATTGCTAAGTCTGAGCAAGAAATAGGCAAAGCAACCG
 Cas9c  (3001) TACCCAAAATTAGAATCTGAATTCGTTTATGGTGACTATAAAGTTTATGATGTACGTAAAATGATTGCTAAATCAGAACAAGAAATTGGTAAAGCTACTG

SpCas9  (3101) CAAAATATTTCTTTTACTCTAATATCATGAACTTCTTCAAAACAGAAATTACACTTGCAAATGGAGAGATTCGCAAACGCCCTCTAATCGAAACTAATGG
 Cas9c  (3101) CTAAATACTTTTTCTATTCAAACATTATGAATTTCTTTAAAACTGAAATTACATTAGCTAACGGTGAAATTCGTAAACGTCCATTAATTGAAACTAATGG

SpCas9  (3201) GGAAACTGGAGAAATTGTCTGGGATAAAGGGCGAGATTTTGCCACAGTGCGCAAAGTATTGTCCATGCCCCAAGTCAATATTGTCAAGAAAACAGAAGTA
 Cas9c  (3201) TGAAACTGGTGAAATTGTATGGGATAAAGGTCGTGATTTCGCTACAGTTCGTAAAGTATTATCAATGCCACAAGTTAATATTGTTAAAAAAACTGAAGTT

SpCas9  (3301) CAGACAGGCGGATTCTCCAAGGAGTCAATTTTACCAAAAAGAAATTCGGACAAGCTTATTGCTCGTAAAAAAGACTGGGATCCAAAAAAATATGGTGGTT
 Cas9c  (3301) CAAACAGGTGGTTTTTCAAAAGAATCTATTTTACCTAAACGTAACTCAGACAAATTAATTGCTCGTAAAAAAGATTGGGATCCTAAAAAATATGGTGGTT

SpCas9  (3401) TTGATAGTCCAACGGTAGCTTATTCAGTCCTAGTGGTTGCTAAGGTGGAAAAAGGGAAATCGAAGAAGTTAAAATCCGTTAAAGAGTTACTAGGGATCAC
 Cas9c  (3401) TCGATTCACCAACAGTAGCTTATTCAGTATTAGTTGTAGCTAAAGTAGAAAAAGGTAAATCTAAAAAATTAAAATCAGTAAAAGAATTATTAGGTATTAC

SpCas9  (3501) AATTATGGAAAGAAGTTCCTTTGAAAAAAATCCGATTGACTTTTTAGAAGCTAAAGGATATAAGGAAGTTAAAAAAGACTTAATCATTAAACTACCTAAA
 Cas9c  (3501) AATTATGGAACGTTCATCATTCGAGAAAAACCCAATTGATTTCTTAGAAGCTAAAGGTTATAAAGAAGTTAAAAAAGATTTAATTATTAAATTACCAAAA

SpCas9  (3601) TATAGTCTTTTTGAGTTAGAAAACGGTCGTAAACGGATGCTGGCTAGTGCCGGAGAATTACAAAAAGGAAATGAGCTGGCTCTGCCAAGCAAATATGTGA
 Cas9c  (3601) TACTCTTTATTTGAATTAGAAAACGGTCGTAAACGTATGTTAGCTTCTGCTGGTGAATTACAAAAAGGTAATGAATTAGCATTACCATCAAAATATGTAA

SpCas9  (3701) ATTTTTTATATTTAGCTAGTCATTATGAAAAGTTGAAGGGTAGTCCAGAAGATAACGAACAAAAACAATTGTTTGTGGAGCAGCATAAGCATTATTTAGA
 Cas9c  (3701) ATTTCTTATACTTAGCTTCACACTACGAAAAATTAAAAGGTTCACCAGAAGATAACGAACAAAAACAATTATTCGTTGAACAACATAAACACTATTTAGA

SpCas9  (3801) TGAGATTATTGAGCAAATCAGTGAATTTTCTAAGCGTGTTATTTTAGCAGATGCCAATTTAGATAAAGTTCTTAGTGCATATAACAAACATAGAGACAAA
 Cas9c  (3801) TGAAATTATTGAACAAATTTCAGAATTTTCAAAACGTGTTATTTTAGCTGATGCTAATTTAGATAAAGTTTTATCTGCTTATAACAAACACCGTGATAAA

SpCas9  (3901) CCAATACGTGAACAAGCAGAAAATATTATTCATTTATTTACGTTGACGAATCTTGGAGCTCCCGCTGCTTTTAAATATTTTGATACAACAATTGATCGTA
 Cas9c  (3901) CCTATTCGTGAACAAGCTGAAAACATTATTCACTTATTTACATTAACAAATTTAGGTGCTCCAGCTGCTTTCAAATATTTCGATACAACAATTGACCGTA

SpCas9  (4001) AACGATATACGTCTACAAAAGAAGTTTTAGATGCCACTCTTATCCATCAATCCATCACTGGTCTTTATGAAACACGCATTGATTTGAGTCAGCTAGGAGG
 Cas9c  (4001) AACGTTACACATCAACAAAAGAAGTTTTAGACGCTACATTAATTCATCAATCAATTACAGGTTTATATGAAACACGTATTGATTTAAGTCAATTAGGTGG

SpCas9  (4101) TGACTGA
 Cas9c  (4101) TGATTAA
```
