# Supplementary material for: Cas9/gRNA-mediated genome editing of yeast mitochondria and Chlamydomonas chloroplasts
Source: PeerJ. 2020 Jan 6;8:e8362. doi: 10.7717/peerj.8362 (PMC6951285; doi:10.7717/peerj.8362)
Supplement: Supplemental Information 3 [file peerj-08-8362-s003.pdf]

SpCas9	(1)	ATGGATAAGAAATACTCAATAGGCTTAGATATCGGCACAAATAGCGTCGGATGGGCGGTGATCACTGATGAATATAAGGTTCCGTCTAAAAAGTTCAAGG
Cas9m	(1)	ATGGATAAAAAATATTCAATCGGTTTAGATATCGGTACAAATTCAGTAGGT**TGA**GCTGTAATCACAGATGAATATAAAGTACCTTCAAAAAAATTTAAAG
SpCas9	(101)	TTCTGGGAAATACAGACCGCCACAGTATCAAAAAAAATCTTATAGGGGCTCTTTTATTTGACAGTGGAGAGACAGCGGAAGCGACTCGTCTCAAACGGAC
Cas9m	(101)	TATTAGGTAATACAGATAGACATTCAATCAAAAAAAATTTAATCGGTGCTTTATTATTTGATTCAGGTGAAACAGCTGAAGCTACAAGATTAAAAAGAAC
SpCas9	(201)	AGCTCGTAGAAGGTATACACGTCGGAAGAATCGTATTTGTTATCTACAGGAGATTTTTTCAAATGAGATGGCGAAAGTAGATGATAGTTTCTTTCATCGA
Cas9m	(201)	AGCTAGAAGAAGATATACAAGAAGAAAAAATAGAATCTGTTATTTACAAGAAATCTTTTCAAATGAAATGGCTAAAGTAGATGATTCATTTTTCCATAGA
SpCas9	(301)	CTTGAAGAGTCTTTTTTGGTGGAAGAAGACAAGAAGCATGAACGTCATCCTATTTTTGGAAATATAGTAGATGAAGTTGCTTATCATGAGAAATATCCAA
Cas9m	(301)	TTAGAAGAATCATTTTTAGTTGAAGAAGATAAAAAACATGAAAGACATCCTATCTTTGGTAATATCGTAGATGAAGTAGCTTATCATGAAAAATATCCTA
SpCas9	(401)	CTATCTATCATCTGCGAAAAAAATTGGTAGATTCTACTGATAAAGCGGATTTGCGCTTAATCTATTTGGCCTTAGCGCATATGATTAAGTTTCGTGGTCA
Cas9m	(401)	CAATCTATCATTTAAGAAAAAAATTAGTAGATTCAACTGATAAAGCTGATTTAAGATTAATCTATTTAGCTTTAGCTCATATGATCAAATTTAGAGGTCA
SpCas9	(501)	TTTTTTGATTGAGGGAGATTTAAATCCTGATAATAGTGATGTGGACAAACTATTTATCCAGTTGGTACAAACCTACAATCAATTATTTGAAGAAAACCCT
Cas9m	(501)	TTTCTTAATCGAAGGTGATTTAAATCCTGATAATTCAGATGTAGATAAATTATTCATCCAATTAGTACAAACATATAATCAATTATTTGAAGAAAATCCT
SpCas9	(601)	ATTAACGCAAGTGGAGTAGATGCTAAAGCGATTCTTTCTGCACGATTGAGTAAATCAAGACGATTAGAAAATCTCATTGCTCAGCTCCCCGGTGAGAAGA
Cas9m	(601)	ATCAATGCTTCAGGTGTAGATGCTAAAGCAATCTTATCAGCTAGATTATCAAAATCAAGAAGATTAGAAAATTTAATCGCTCAATTACCTGGAGAAAAAA
SpCas9	(701)	AAAATGGCTTATTTGGGAATCTCATTGCTTTGTCATTGGGTTTGACCCCTAATTTTAAATCAAATTTTGATTTGGCAGAAGATGCTAAATTACAGCTTTC
Cas9m	(701)	AAAATGGTTTATTTGGTAATTTAATCGCATTATCATTAGGTTTAACTCCTAATTTCAAATCAAATTTCGATTTAGCTGAAGATGCAAAATTACAATTATC
SpCas9	(801)	AAAAGATACTTACGATGATGATTTAGATAATTTATTGGCGCAAATTGGAGATCAATATGCTGATTTGTTTTTGGCAGCTAAGAATTTATCAGATGCTATT
Cas9m	(801)	TAAAGATACATATGATGATGATTTAGATAATTTATTAGCTCAAATCGGTGATCAATATGCTGATTTATTCTTAGCTGCTAAAAATTTATCAGATGCTATC
SpCas9	(901)	TTACTTTCAGATATCCTAAGAGTAAATACTGAAATAACTAAGGCTCCCCTATCAGCTTCAATGATTAAACGCTACGATGAACATCATCAAGACTTGACTC
Cas9m	(901)	TTATTATCAGATATCTTAAGAGTAAATACAGAAATCACAAAAGCACCTTTATCAGCTTCAATGATCAAAAGATATGATGAACATCATCAAGATTTAACAT
SpCas9	(1001)	TTTTAAAAGCTTTAGTTCGACAACAACTTCCAGAAAAGTATAAAGAAATCTTTTTTGATCAATCAAAAAACGGATATGCAGGTTATATTGATGGGGGAGC
Cas9m	(1001)	TATTAAAAGCTTTAGTAAGACAACAATTACCAGAAAAATATAAAGAAATCTTCTTTGATCAATCAAAAAATGGTTATGCTGGTTATATCGATGGTGGTGC
SpCas9	(1101)	TAGCCAAGAAGAATTTTATAAATTTATCAAACCAATTTTAGAAAAAATGGATGGTACTGAGGAATTATTGGTGAAACTAAATCGTGAAGATTTGCTGCGC
Cas9m	(1101)	TTCTCAAGAAGAATTCTATAAATTCATCAAACCTATCTTAGAAAAAATGGATGGTACAGAAGAATTATTAGTAAAATTAAATAGAGAAGATTTATTAAGA
SpCas9	(1201)	AAGCAACGGACCTTTGACAACGGCTCTATTCCCCATCAAATTCACTTGGGTGAGCTGCATGCTATTTTGAGAAGACAAGAAGACTTTTATCCATTTTTAA
Cas9m	(1201)	AAACAAAGAACATTTGATAATGGTTCAATCCCTCATCAAATCCATTTAGGTGAATTACATGCAATCTTAAGAAGACAAGAAGATTTTTATCCTTTCTTAA
SpCas9	(1301)	AAGACAATCGTGAGAAGATTGAAAAAATCTTGACTTTTCGAATTCCTTATTATGTTGGTCCATTGGCGCGTGGCAATAGTCGTTTTGCATGGATGACTCG
Cas9m	(1301)	AAGATAATAGAGAAAAAATCGAAAAAATCTTAACATTTAGAATCCCTTATTATGTAGGTCCTTTAGCTAGAGGTAATTCAAGATTTGCT**TGA**ATGACAAG
SpCas9	(1401)	GAAGTCTGAAGAAACAATTACCCCATGGAATTTTGAAGAAGTTGTCGATAAAGGTGCTTCAGCTCAATCATTTATTGAACGCATGACAAACTTTGATAAA
Cas9m	(1401)	AAAATCAGAAGAAACAATCACACCTTGGAATTTTGAAGAAGTAGTAGATAAAGGAGCTTCAGCACAATCATTTATCGAAAGAATGACAAATTTTGATAAA
SpCas9	(1501)	AATCTTCCAAATGAAAAAGTACTACCAAAACATAGTTTGCTTTATGAGTATTTTACGGTTTATAACGAATTGACAAAGGTCAAATATGTTACTGAAGGAA
Cas9m	(1501)	AATTTACCTAATGAAAAAGTTTTACCTAAACATTCATTATTATATGAATATTTCACAGTATATAATGAATTAACAAAAGTAAAATATGTAACAGAAGGTA
SpCas9	(1601)	TGCGAAAACCAGCATTTCTTTCAGGTGAACAGAAGAAAGCCATTGTTGATTTACTCTTCAAAACAAATCGAAAAGTAACCGTTAAGCAATTAAAAGAAGA
Cas9m	(1601)	TGAGAAAACCTGCTTTTTTATCAGGTGAACAAAAAAAAGCAATCGTAGATTTATTATTTAAAACAAATAGAAAAGTAACAGTAAAACAATTAAAAGAAGA
SpCas9	(1701)	TTATTTCAAAAAAATAGAATGTTTTGATAGTGTTGAAATTTCAGGAGTTGAAGATAGATTTAATGCTTCATTAGGTACCTACCATGATTTGCTAAAAATT
Cas9m	(1701)	TTATTTCAAAAAAATCGAATGTTTTGATTCAGTAGAAATCTCTGGTGTAGAAGATAGATTTAATGCTTCTTTAGGTACATATCATGATTTATTAAAAATC
SpCas9	(1801)	ATTAAAGATAAAGATTTTTTGGATAATGAAGAAAATGAAGATATCTTAGAGGATATTGTTTTAACATTGACCTTATTTGAAGATAGGGAGATGATTGAGG
Cas9m	(1801)	ATCAAAGATAAAGATTTCTTAGATAATGAAGAAAATGAAGATATCTTAGAAGATATCGTATTAACATTAACTTTATTCGAAGATAGAGAAATGATCGAAG
SpCas9	(1901)	AAAGACTTAAAACATATGCTCACCTCTTTGATGATAAGGTGATGAAACAGCTTAAACGTCGCCGTTATACTGGTTGGGGACGTTTGTCTCGAAAATTGAT
Cas9m	(1901)	AAAGATTAAAAACATATGCTCATTTATTTGATGATAAAGTAATGAAACAATTAAAAAGAAGAAGATATACTGGT**TGA**GGTAGATTATCAAGAAAATTAAT
SpCas9	(2001)	TAATGGTATTAGGGATAAGCAATCTGGCAAAACAATATTAGATTTTTTGAAATCAGATGGTTTTGCCAATCGCAATTTTATGCAGCTGATCCATGATGAT
Cas9m	(2001)	CAATGGTATCAGAGATAAACAATCTGGTAAAACAATCTTAGATTTCTTAAAATCAGATGGTTTTGCTAATAGAAATTTCATGCAATTAATCCATGATGAT
SpCas9	(2101)	AGTTTGACATTTAAAGAAGACATTCAAAAAGCACAAGTGTCTGGACAAGGCGATAGTTTACATGAACATATTGCAAATTTAGCTGGTAGCCCTGCTATTA
Cas9m	(2101)	AGTTTAACTTTTAAAGAAGATATCCAAAAAGCTCAAGTATCAGGTCAAGGTGATTCATTACATGAACATATCGCTAATTTAGCTGGTTCTCCTGCTATCA
SpCas9	(2201)	AAAAAGGTATTTTACAGACTGTAAAAGTTGTTGATGAATTGGTCAAAGTAATGGGGCGGCATAAGCCAGAAAATATCGTTATTGAAATGGCACGTGAAAA
Cas9m	(2201)	AAAAAGGTATCTTACAAACTGTAAAAGTTGTAGATGAATTAGTTAAAGTTATGGGTAGACATAAACCTGAAAATATCGTAATCGAAATGGCAAGAGAAAA
SpCas9	(2301)	TCAGACAACTCAAAAGGGCCAGAAAAATTCGCGAGAGCGTATGAAACGAATCGAAGAAGGTATCAAAGAATTAGGAAGTCAGATTCTTAAAGAGCATCCT
Cas9m	(2301)	TCAAACAACACAAAAAGGACAAAAAAATTCAAGAGAAAGAATGAAAAGAATCGAAGAAGGTATCAAAGAATTAGGTTCACAAATCTTAAAAGAACATCCT
SpCas9	(2401)	GTTGAAAATACTCAATTGCAAAATGAAAAGCTCTATCTCTATTATCTCCAAAATGGAAGAGACATGTATGTGGACCAAGAATTAGATATTAATCGTTTAA
Cas9m	(2401)	GTAGAAAATACACAATTACAAAATGAAAAATTATATTTATATTATTTACAAAATGGTAGAGATATGTATGTAGATCAAGAATTAGATATCAATAGATTAT
SpCas9	(2501)	GTGATTATGATGTCGATCACATTGTTCCACAAAGTTTCCTTAAAGACGATTCAATAGACAATAAGGTCTTAACGCGTTCTGATAAAAATCGTGGTAAATC
Cas9m	(2501)	CTGATTATGATGTAGATCATATCGTACCTCAATCATTCTTAAAAGATGATTCAATCGATAATAAAGTATTAACAAGATCAGATAAAAATAGAGGTAAAAG
SpCas9	(2601)	GGATAACGTTCCAAGTGAAGAAGTAGTCAAAAAGATGAAAAACTATTGGAGACAACTTCTAAACGCCAAGTTAATCACTCAACGTAAGTTTGATAATTTA
Cas9m	(2601)	TGATAATGTACCTTCTGAAGAAGTTGTAAAAAAAATGAAAAATTAT**TGA**AGACAATTATTAAATGCTAAATTAATCACACAAAGAAAATTCGATAATTTA
SpCas9	(2701)	ACGAAAGCTGAACGTGGAGGTTTGAGTGAACTTGATAAAGCTGGTTTTATCAAACGCCAATTGGTTGAAACTCGCCAAATCACTAAGCATGTGGCACAAA
Cas9m	(2701)	ACAAAAGCTGAAAGAGGTGGTTTATCAGAATTAGATAAAGCTGGTTTCATCAAAAGACAATTAGTTGAAACAAGACAAATCACTAAACATGTTGCTCAAA
SpCas9	(2801)	TTTTGGATAGTCGCATGAATACTAAATACGATGAAAATGATAAACTTATTCGAGAGGTTAAAGTGATTACCTTAAAATCTAAATTAGTTTCTGACTTCCG
Cas9m	(2801)	TCTTAGATAGTAGAATGAATACAAAATATGATGAAAATGATAAATTAATCAGAGAAGTAAAAGTAATCACATTAAAATCTAAATTAGTATCAGATTTTAG
SpCas9	(2901)	AAAAGATTTCCAATTCTATAAAGTACGTGAGATTAACAATTACCATCATGCCCATGATGCGTATCTAAATGCCGTCGTTGGAACTGCTTTGATTAAGAAA
Cas9m	(2901)	AAAAGATTTTCAATTCTATAAAGTAAGAGAAATCAATAATTATCATCATGCTCATGATGCTTATTTAAATGCTGTAGTAGGTACAGCTTTAATCAAAAAA
SpCas9	(3001)	TATCCAAAACTTGAATCGGAGTTTGTCTATGGTGATTATAAAGTTTATGATGTTCGTAAAATGATTGCTAAGTCTGAGCAAGAAATAGGCAAAGCAACCG

Cas9m (3001) TATCCAAAATTAGAATCAGAATTTGTATATGGAGATTATAAAGTATATGATGTTAGAAAAATGATCGCTAAATCAGAACAAGAAATCGGTAAAGCTACTG

SpCas9 (3101) CAAAATATTTCTTTTACTCTAATATCATGAACTTCTTCAAAACAGAAATTACACTTGCAAATGGAGAGATTCGCAAACGCCCTCTAATCGAAACTAATGG
Cas9m (3101) CTAAATATTTCTTTTATTCAAATATCATGAATTTTTTCAAAACTGAAATCACTTTAGCTAATGGTGAAATCAGAAAAAGACCTTTAATCGAAACAAATGG
SpCas9 (3201) GGAAACTGGAGAAATTGTCTGGGATAAAGGGCGAGATTTTGCCACAGTGCGCAAAGTATTGTCCATGCCCCAAGTCAATATTGTCAAGAAAACAGAAGTA
Cas9m (3201) TGAAACTGGTGAAATCGTA**TGA**GATAAAGGTAGAGATTTTGCTACAGTAAGAAAAGTATTATCAATGCCTCAAGTAAATATCGTTAAAAAAACTGAAGTA

SpCas9 (3301) CAGACAGGCGGATTCTCCAAGGAGTCAATTTTACCAAAAAGAAATTCGGACAAGCTTATTGCTCGTAAAAAAGACTGGGATCCAAAAAAATATGGTGGTT
Cas9m (3301) CAAACTGGTGGTTTTTCTAAAGAATCAATCTTACCAAAAAGAAATTCAGATAAATTAATCGCTAGAAAAAAAGAT**TGA**GATCCAAAAAAATATGGTGGTT

SpCas9 (3401) TTGATAGTCCAACGGTAGCTTATTCAGTCCTAGTGGTTGCTAAGGTGGAAAAAGGGAAATCGAAGAAGTTAAAATCCGTTAAAGAGTTACTAGGGATCAC
Cas9m (3401) TCGATTCACCTACAGTAGCATATTCAGTATTAGTAGTAGCAAAAGTAGAAAAAGGTAAATCTAAAAAATTAAAATCAGTAAAAGAATTATTAGGTATCAC

SpCas9 (3501) AATTATGGAAAGAAGTTCCTTTGAAAAAAATCCGATTGACTTTTTAGAAGCTAAAGGATATAAGGAAGTTAAAAAAGACTTAATCATTAAACTACCTAAA
Cas9m (3501) AATCATGGAAAGATCATCATTCGAAAAAAATCCAATCGATTTTTTAGAAGCTAAAGGTTATAAAGAAGTTAAAAAAGATTTAATCATCAAATTACCTAAA

SpCas9 (3601) TATAGTCTTTTTGAGTTAGAAAACGGTCGTAAACGGATGCTGGCTAGTGCCGGAGAATTACAAAAAGGAAATGAGCTGGCTCTGCCAAGCAAATATGTGA
Cas9m (3601) TATAGTTTATTTGAATTAGAAAATGGAAGAAAAAGAATGTTAGCATCAGCTGGTGAATTACAAAAAGGTAATGAATTAGCATTACCATCTAAATATGTTA

SpCas9 (3701) ATTTTTTATATTTAGCTAGTCATTATGAAAAGTTGAAGGGTAGTCCAGAAGATAACGAACAAAAACAATTGTTTGTGGAGCAGCATAAGCATTATTTAGA
Cas9m (3701) ATTTCTTATATTTAGCATCACATTATGAAAAATTAAAAGGTTCTCCTGAAGATAATGAACAAAAACAATTATTTGTAGAACAACATAAACATTATTTAGA

SpCas9 (3801) TGAGATTATTGAGCAAATCAGTGAATTTTCTAAGCGTGTTATTTTAGCAGATGCCAATTTAGATAAAGTTCTTAGTGCATATAACAAACATAGAGACAAA
Cas9m (3801) TGAAATCATCGAACAAATCTCAGAATTTTCAAAAAGAGTAATCTTAGCAGATGCAAATTTAGATAAAGTTTTATCTGCTTATAATAAACATAGAGATAAA

SpCas9 (3901) CCAATACGTGAACAAGCAGAAAATATTATTCATTTATTTACGTTGACGAATCTTGGAGCTCCCGCTGCTTTTAAATATTTTGATACAACAATTGATCGTA
Cas9m (3901) CCTATCAGAGAACAAGCAGAAAATATCATCCATTTATTCACATTAACAAATTTAGGTGCTCCTGCTGCTTTCAAATATTTCGATACAACAATCGATAGAA

SpCas9 (4001) AACGATATACGTCTACAAAAGAAGTTTTAGATGCCACTCTTATCCATCAATCCATCACTGGTCTTTATGAAACACGCATTGATTTGAGTCAGCTAGGAGG
Cas9m (4001) AAAGATATACTTCAACAAAAGAAGTATTAGATGCAACATTAATCCATCAATCAATCACAGGTTTATATGAAACTAGAATCGATTTATCTCAATTAGGTGG

SpCas9 (4101) TGACTGA
Cas9m (4101) TGATTAA
