# Supplementary figures and images for: Cas9/gRNA-mediated genome editing of yeast mitochondria and Chlamydomonas chloroplasts
Source: PeerJ. 2020 Jan 6;8:e8362. doi: 10.7717/peerj.8362 (PMC6951285; doi:10.7717/peerj.8362)

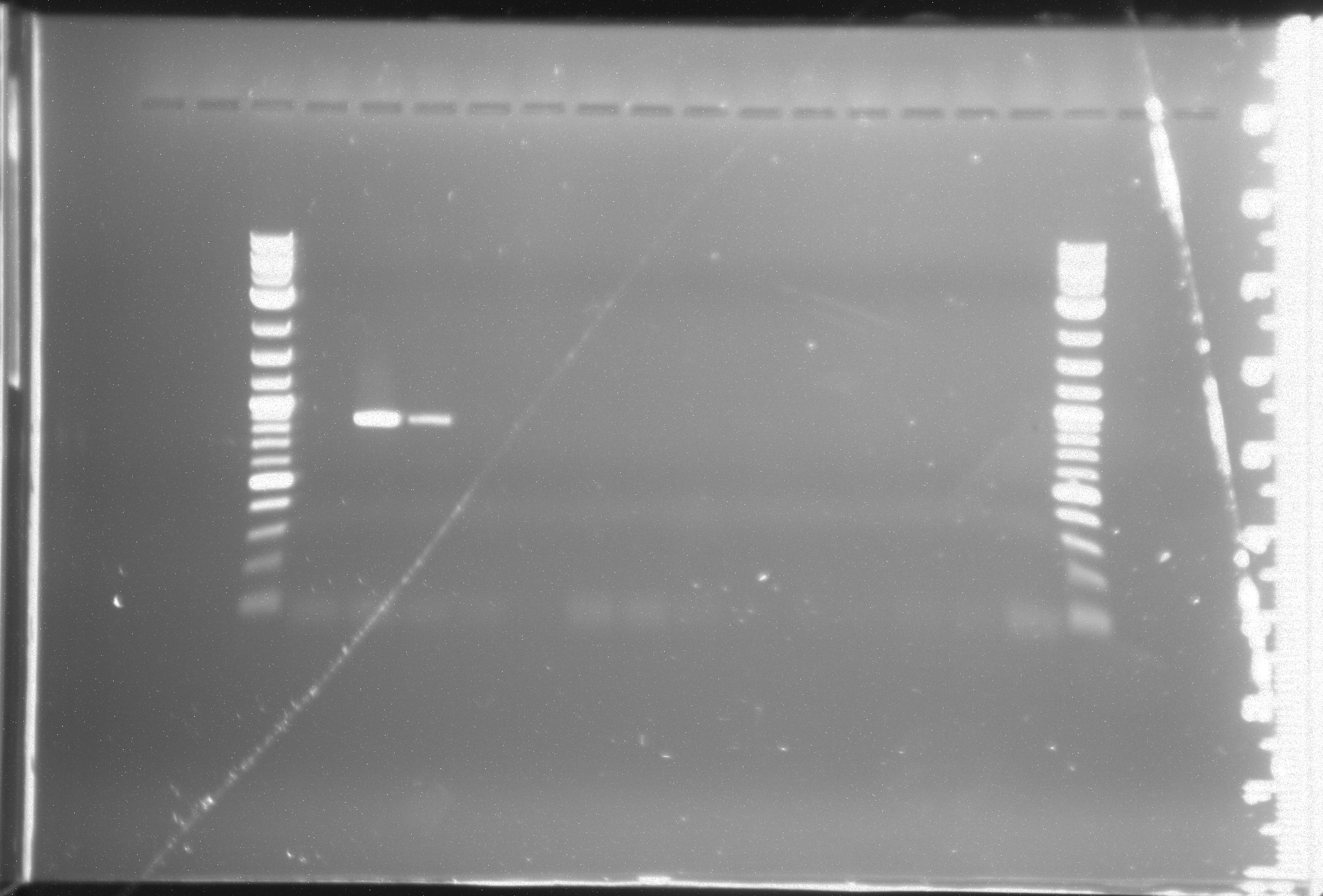

Supplement: Supplemental Information 4 [file peerj-08-8362-s004.tif]

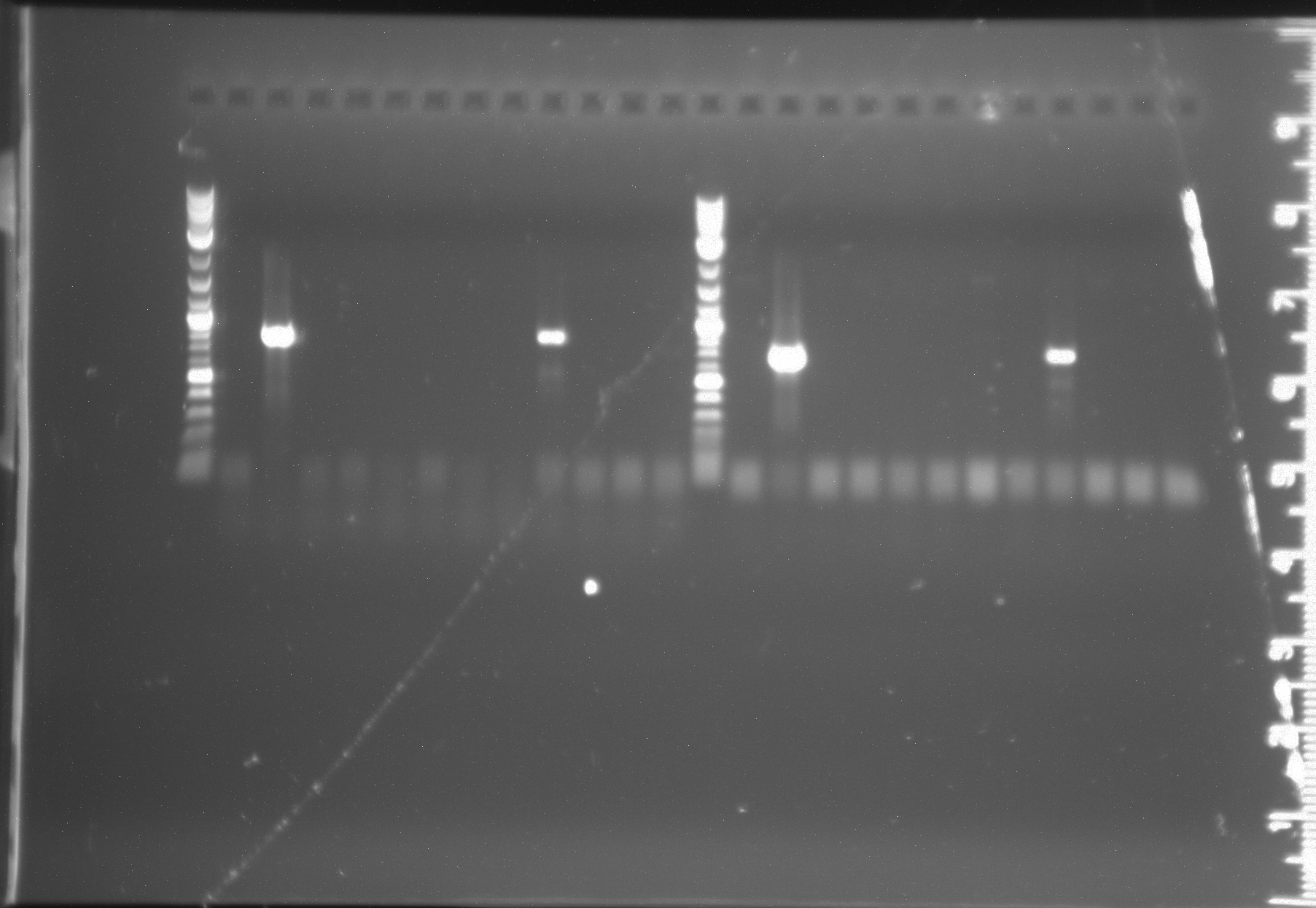

Supplement: Supplemental Information 5 [file peerj-08-8362-s005.tif]

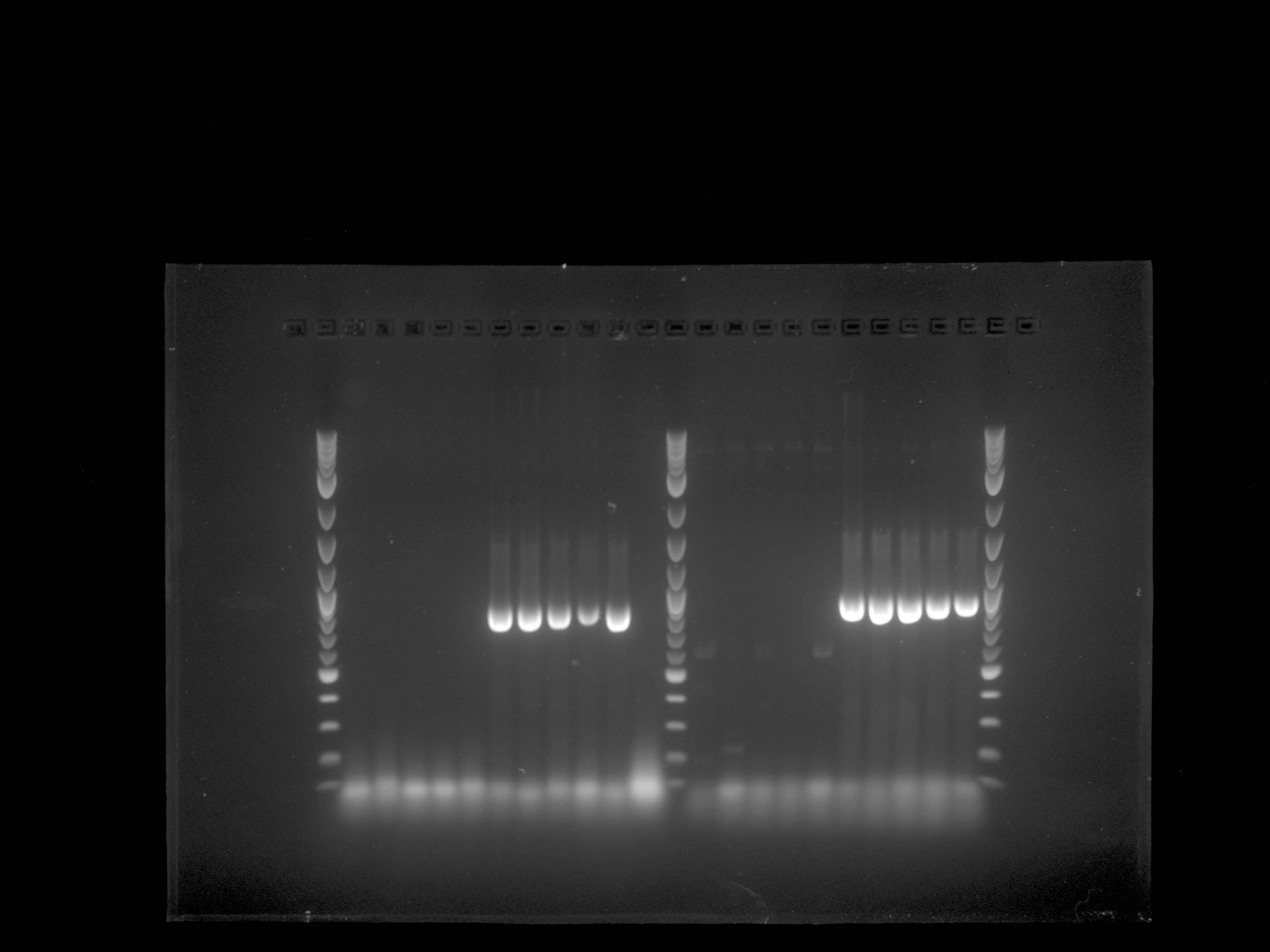

Supplement: Supplemental Information 6 — The right half of the gel image corresponds to the one presented in Fig. 5B. [file peerj-08-8362-s006.tif]

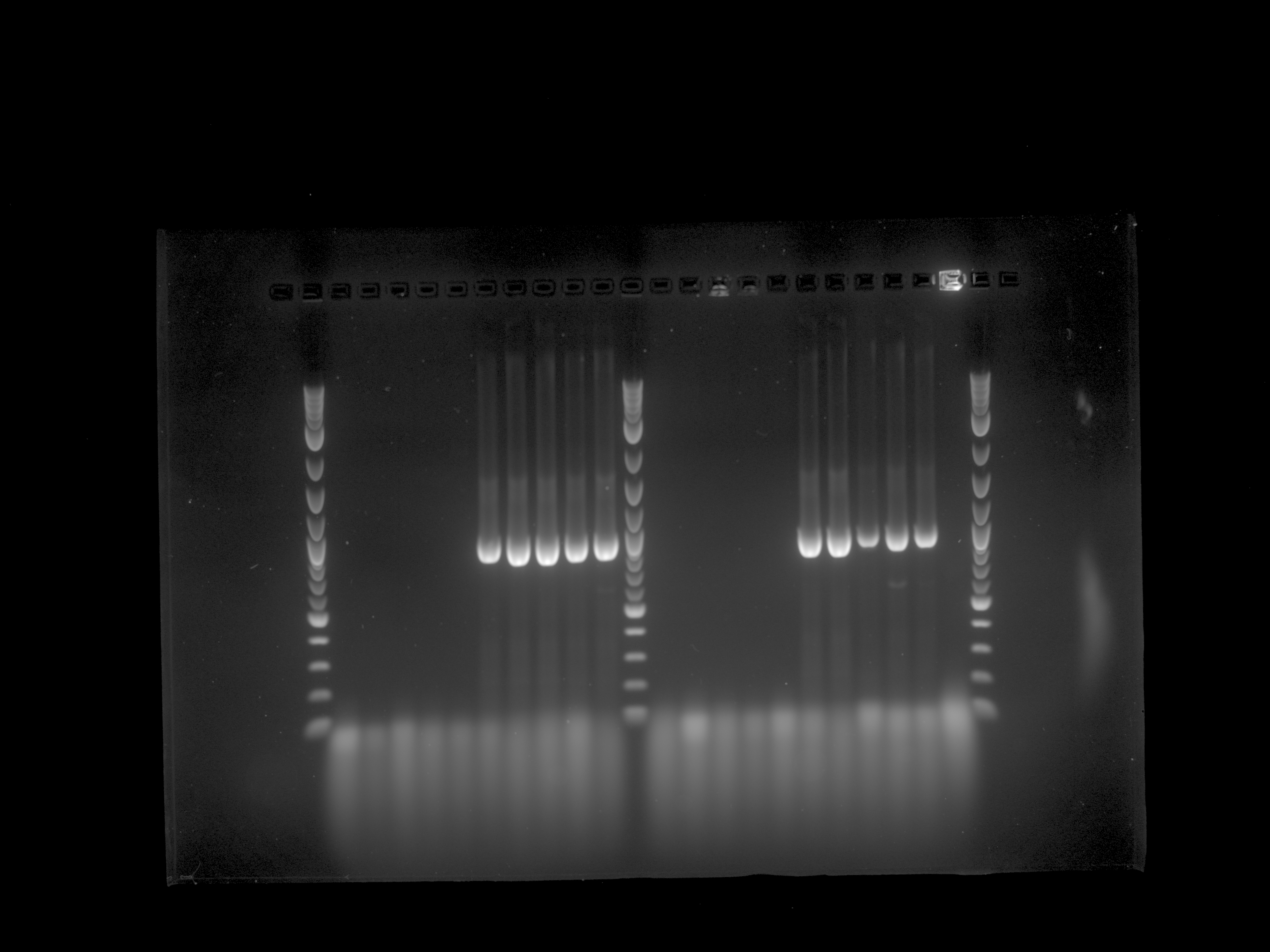

Supplement: Supplemental Information 7 — The left half of the gel image corresponds to the one presented in Fig. 5B. [file peerj-08-8362-s007.tif]

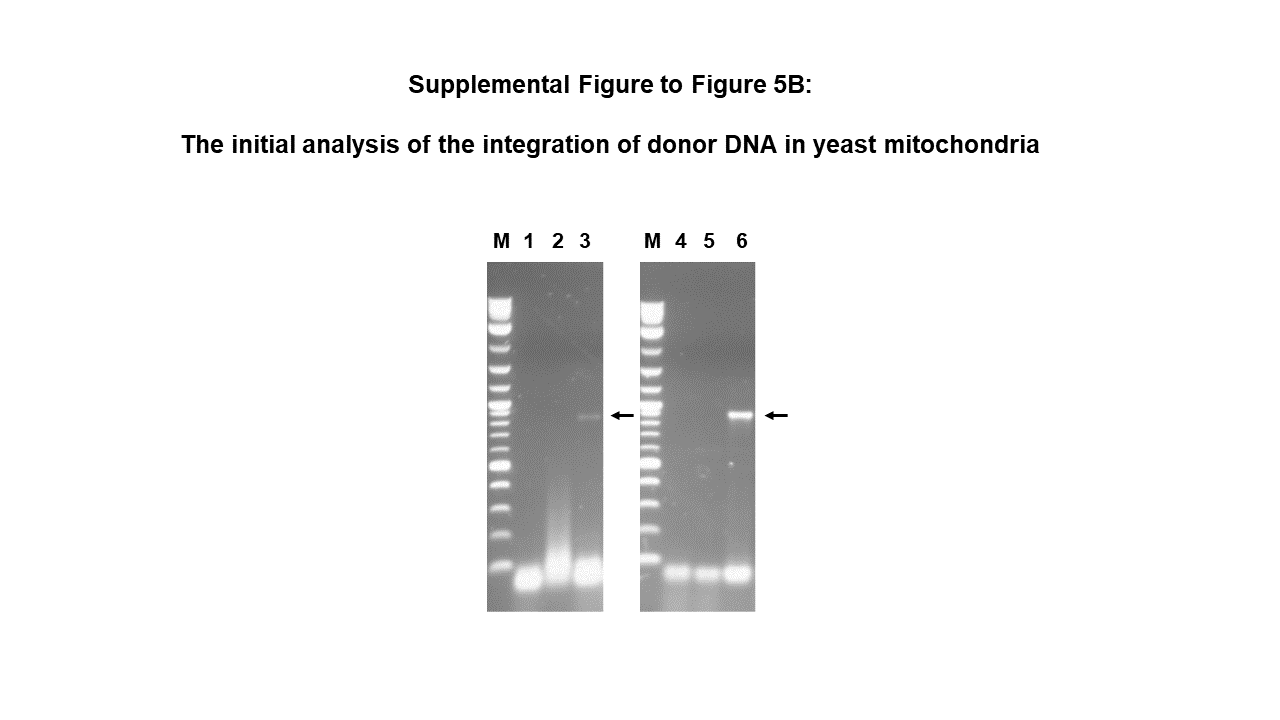

Supplement: Supplemental Information 8 — PCR amplification of the junction regions of the replaced DNA. Lanes 1-–3 for the left junction amplified with primers C and 12. Lanes 4–6 for the right junction amplified with primers F and 11. Control samples derived from HS6 are in lanes 1, 2, 4 and 5. Samples derived from HS8 are in lanes 3 and 6. The size expected from the deduced sequence of DNA replacement is indicated by arrows (C/12 amplicon: 870 bp, F/11 amplicon: 907 bp). (M) 1 kb plus molecular weight marker (New England Biolabs, Ipswich, MA, USA). [file peerj-08-8362-s008.png]

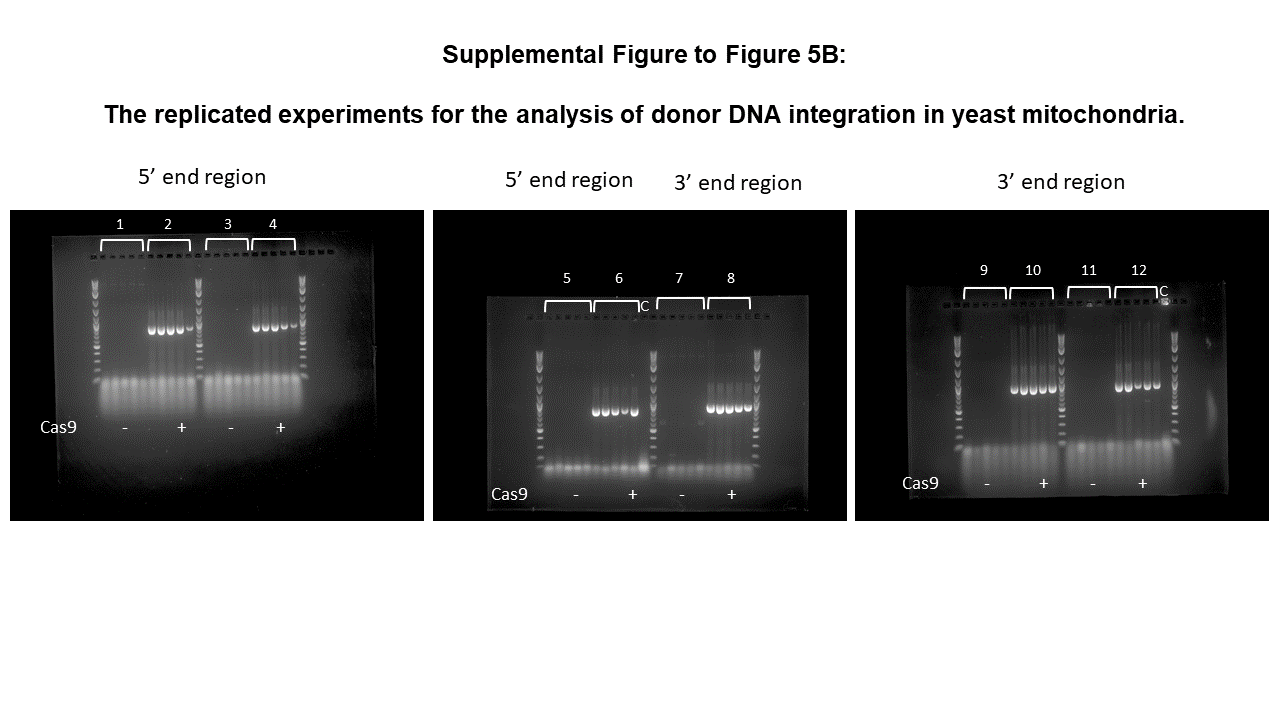

Supplement: Supplemental Information 9 — Experiment sets 1–6: PCR amplification of the 5′ region of the integrated donor DNA using primers C and 12; experimental sets 7–12: PCR amplification of the 3′ region of the integrated donor DNA using primers F and 11 (see Fig. 5B legend and Materials and Methods). Sets 1, 3, 5, 7, 9 and 11 represent the analysis of the yeast strain carrying the control Edit Plasmid HS6. In each of these sets, the order of the samples is #1–#5, identical to that of Fig. 5B. Sets 2, 4, 6, 8, 10 and 12 represent the analysis of the yeast strain carrying the Edit Plasmid HS8 with active Cas9/gRNA. In each of these sets, the order of the samples is #6–#10, identical to that of Fig. 5B. (C) Wild-type CUY563 strain; 1 kb plus molecular weight marker (New England Biolabs, Ipswich, MA, USA) are used for DNA size reference. All images are raw gel images. [file peerj-08-8362-s009.png]

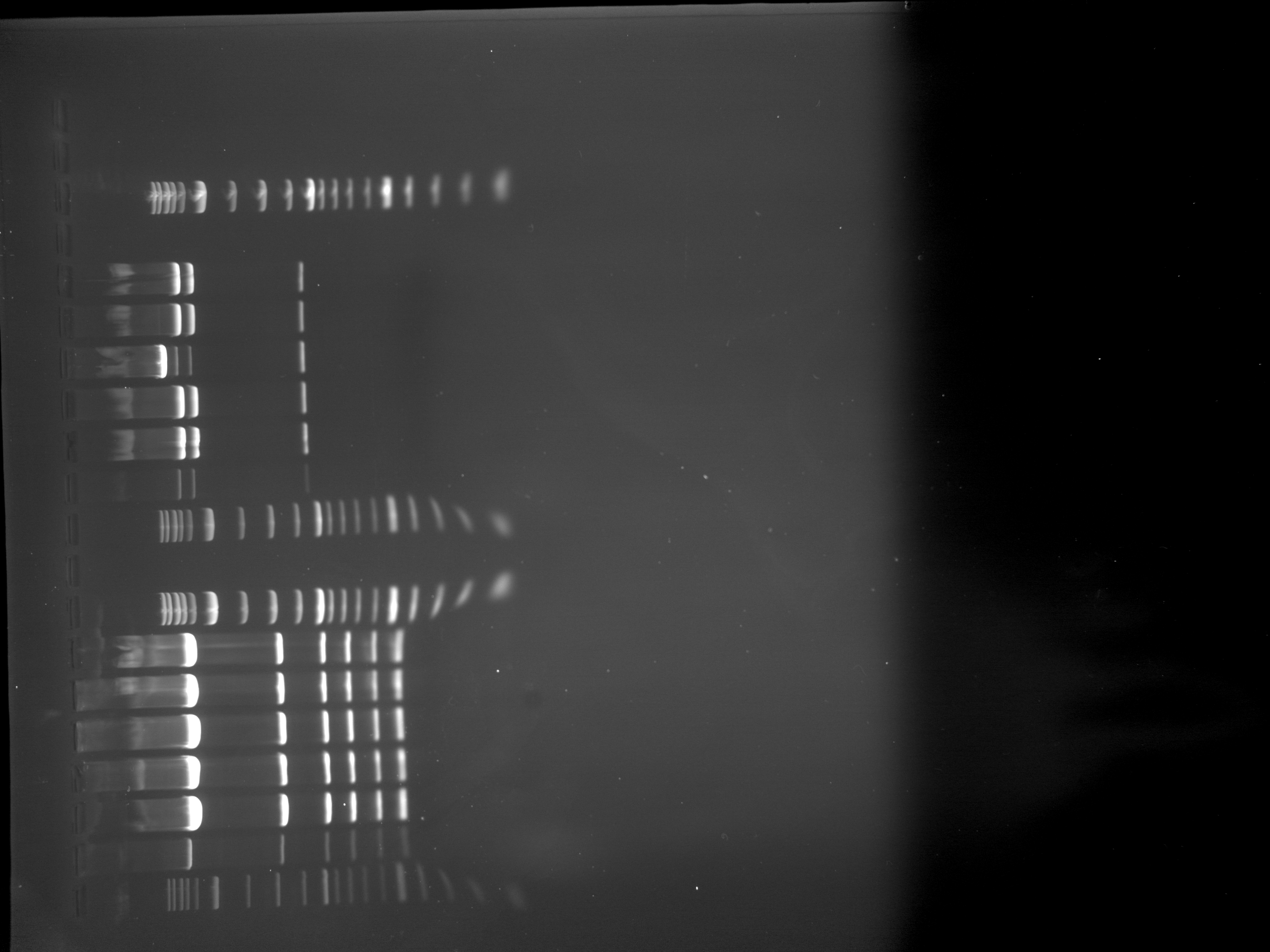

Supplement: Supplemental Information 10 [file peerj-08-8362-s010.tif]

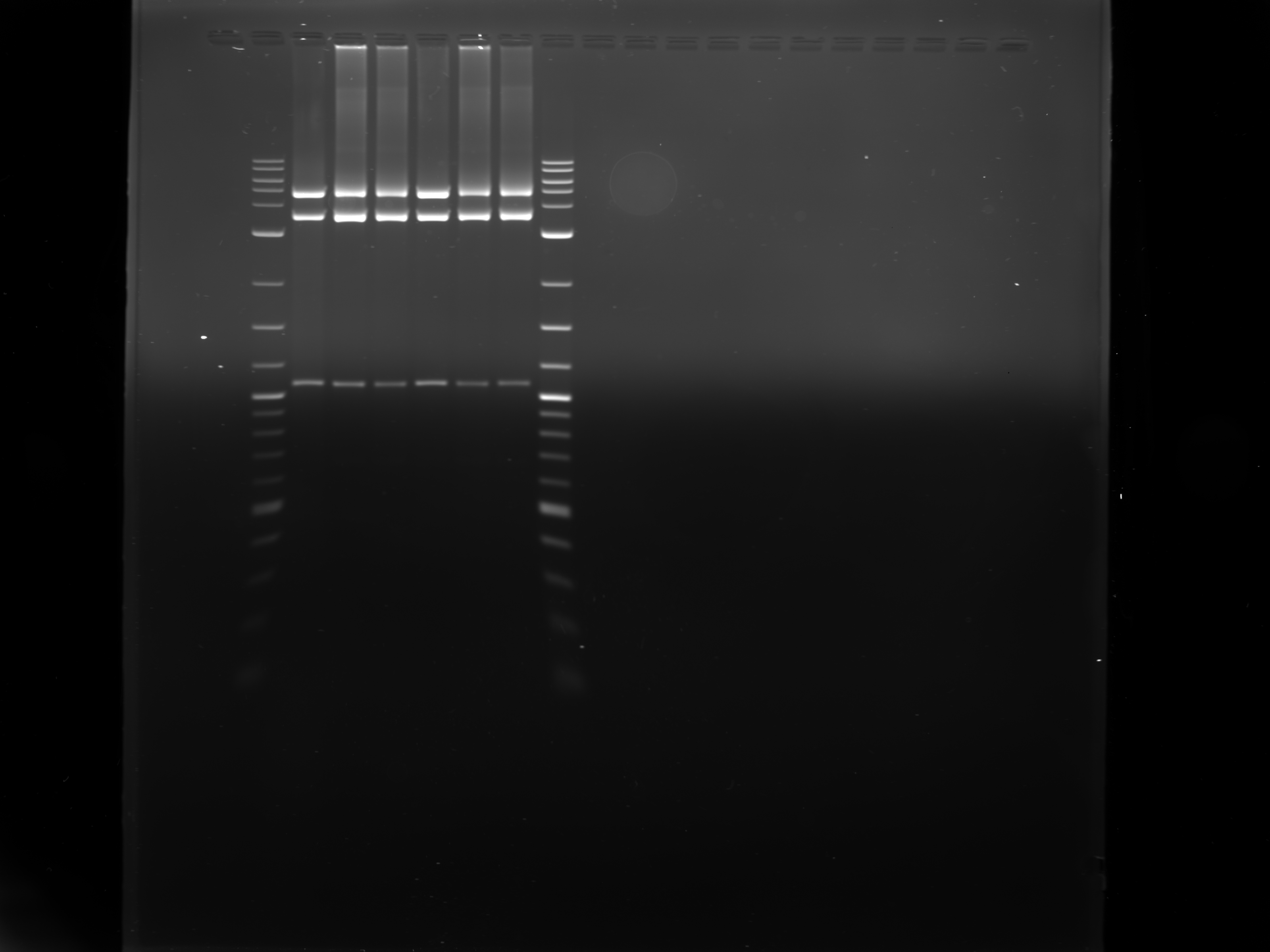

Supplement: Supplemental Information 11 [file peerj-08-8362-s011.tif]

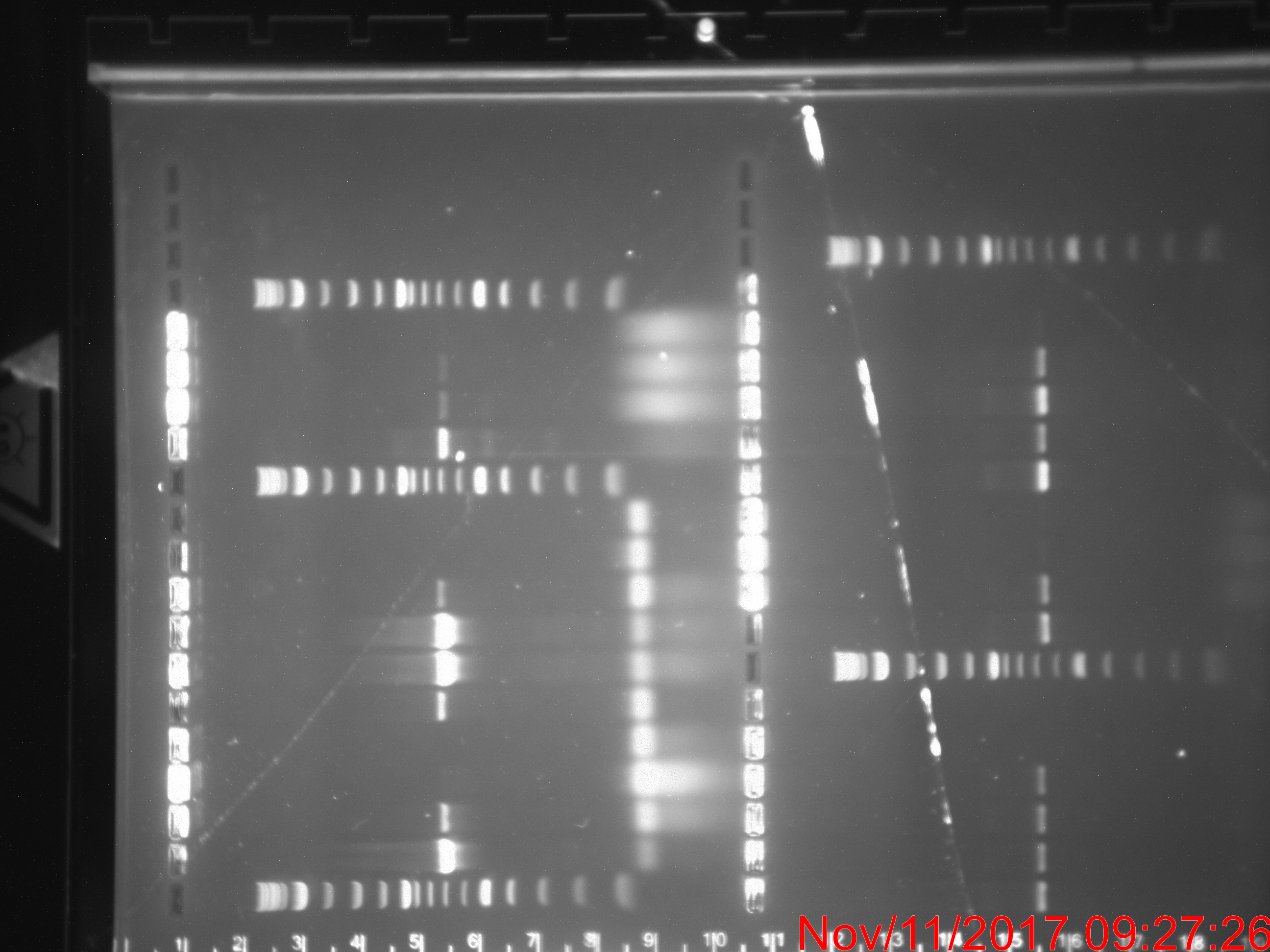

Supplement: Supplemental Information 12 — The lanes 1–5 of the upper part of the gel correspond to the Fig. 6C. [file peerj-08-8362-s012.tif]

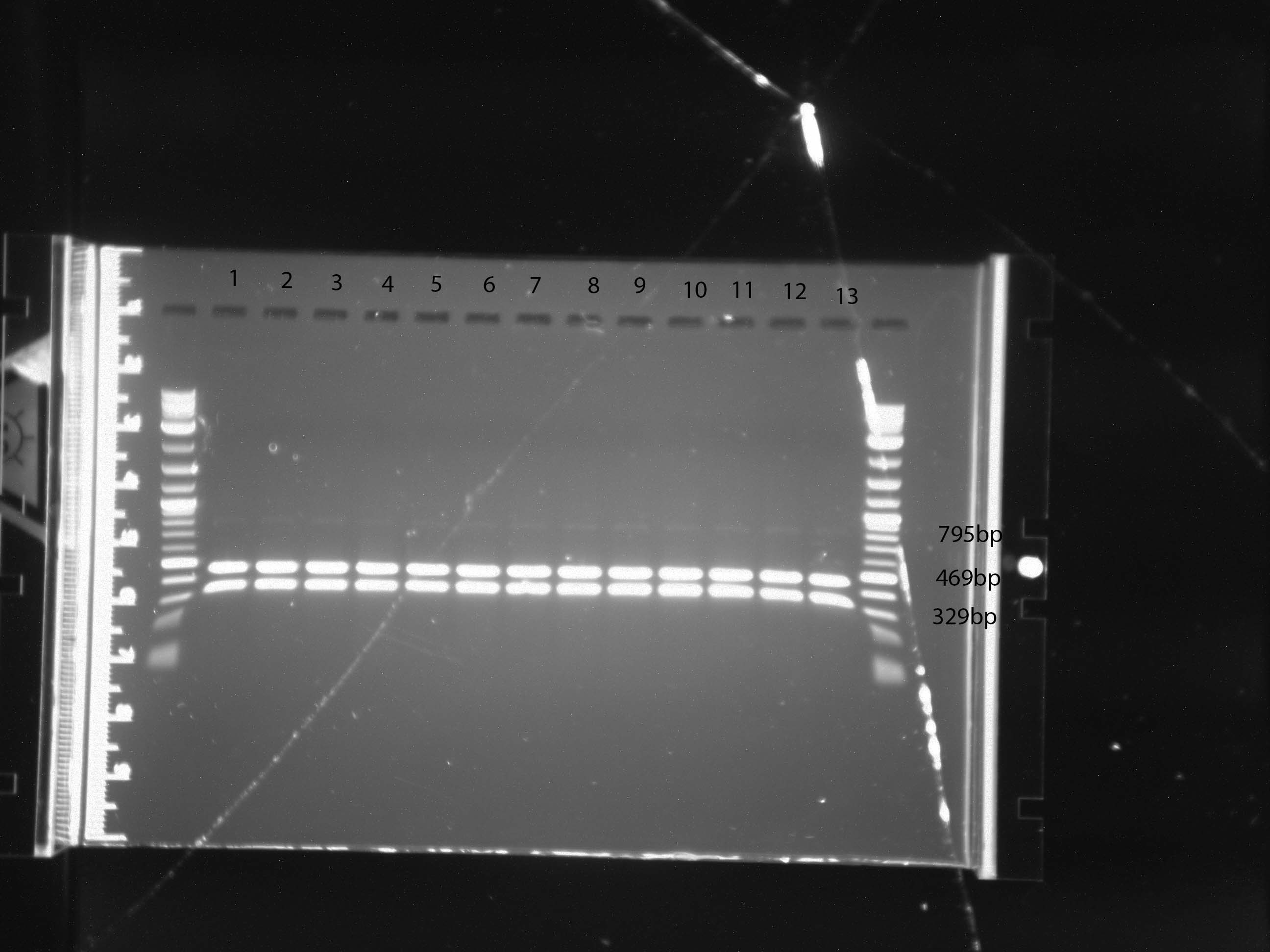

Supplement: Supplemental Information 23 — The DNA region covering one of the Cas9/gRNA target sites was amplified from the cells carrying Chloroplasts transformed with Edit Plasmids, digested with the AvaII restriction enzyme and separated on an agarose gel. Lane 1 and 2: YP5 transformants; lane 3–7: YP11 transformants; lane 8: YP29; lane 9: YP30; lane 10–11: YP31; lane 12: YP33; and lane 13: Untransformed cells. 1 kb Plus Molecular Weight Marker (New England Biolabs, Ipswich, MA, USA) on outer lanes (see Materials and Methods). [file peerj-08-8362-s023.jpg]
