# Supplementary material for: Cas9/gRNA-mediated genome editing of yeast mitochondria and Chlamydomonas chloroplasts
Source: PeerJ. 2020 Jan 6;8:e8362. doi: 10.7717/peerj.8362 (PMC6951285; doi:10.7717/peerj.8362)
Supplement: Supplemental Information 13 — The randomly selected clones derived from various constructs were digested with AvaII and separated on agarose gel. This raw image corresponds to the Step 7 (see Materials and Methods). Clones that had undigested DNA (labeled with *) were sequenced subsequently. [file peerj-08-8362-s013.pptx]

## Slide 1
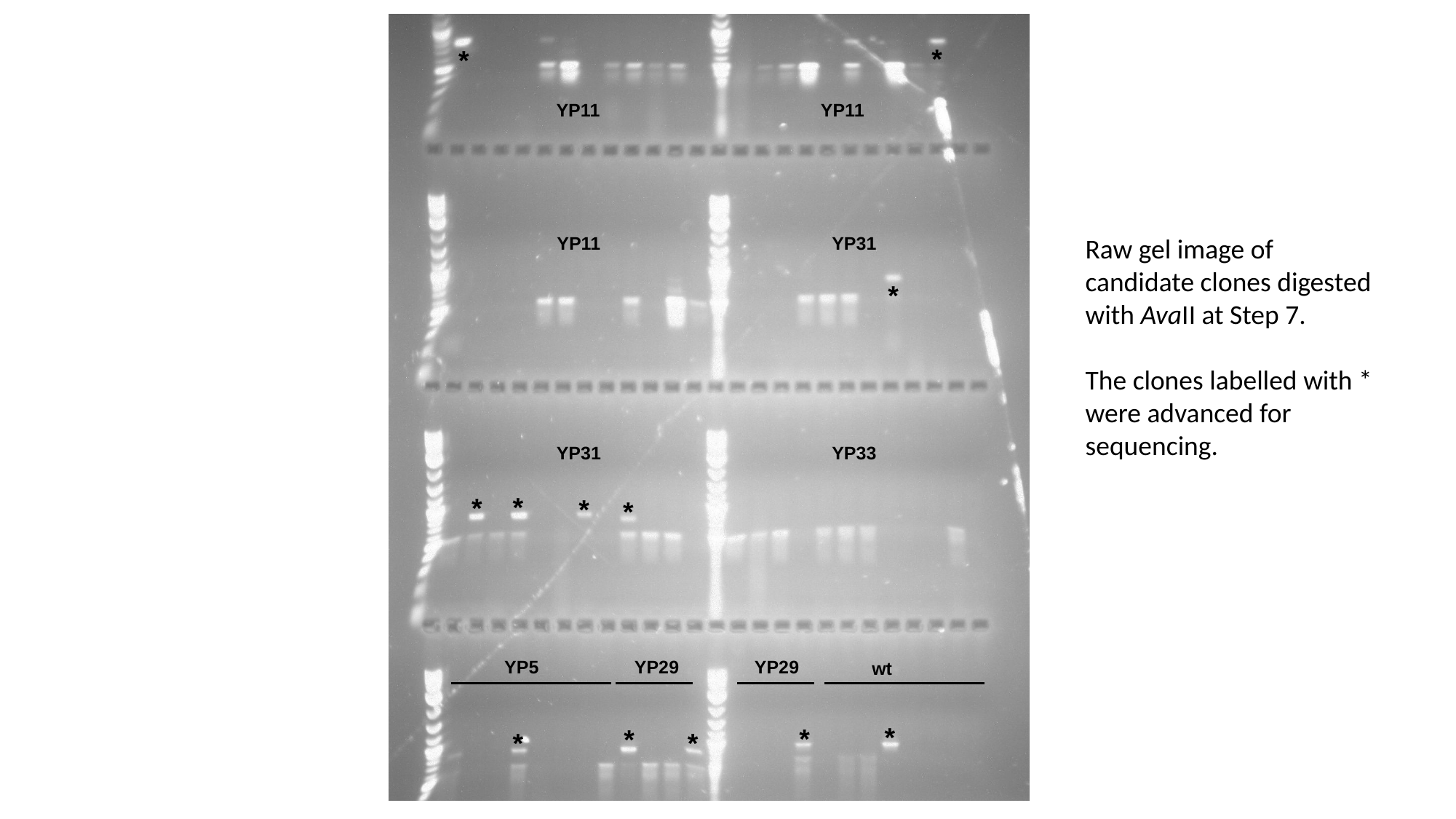

*
*
YP11
YP11
YP11
YP31
*
YP33
YP31
*
*
*
*
YP29
YP29
YP5
wt
*
*
*
*
*
Raw gel image of candidate clones digested with AvaII at Step 7.
The clones labelled with * were advanced for sequencing.
