# Supplementary material for: Cas9/gRNA-mediated genome editing of yeast mitochondria and Chlamydomonas chloroplasts
Source: PeerJ. 2020 Jan 6;8:e8362. doi: 10.7717/peerj.8362 (PMC6951285; doi:10.7717/peerj.8362)
Supplement: Supplemental Information 18 — Raw gel images are shown in this file along with the sequence information, which are summarized in Fig. 3 and Table 4. [file peerj-08-8362-s018.pptm]

## Slide 1
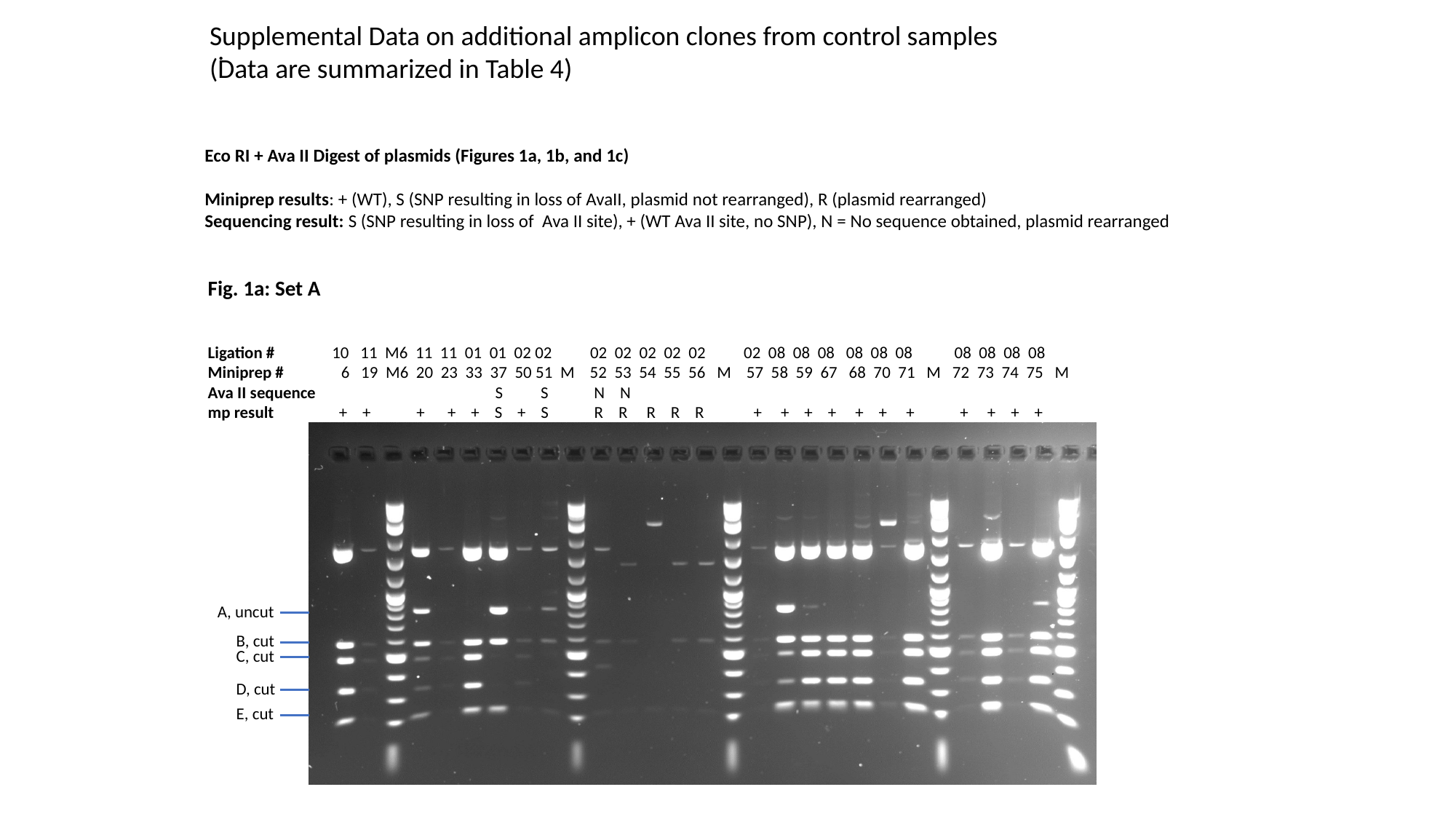

Supplemental Data on additional amplicon clones from control samples
(Data are summarized in Table 4)
.
Eco RI + Ava II Digest of plasmids (Figures 1a, 1b, and 1c)
Miniprep results: + (WT), S (SNP resulting in loss of AvaII, plasmid not rearranged), R (plasmid rearranged)
Sequencing result: S (SNP resulting in loss of Ava II site), + (WT Ava II site, no SNP), N = No sequence obtained, plasmid rearranged
Fig. 1a: Set A
Ligation # 10 11 M6 11 11 01 01 02 02 02 02 02 02 02 02 08 08 08 08 08 08 08 08 08 08
Miniprep # 6 19 M6 20 23 33 37 50 51 M 52 53 54 55 56 M 57 58 59 67 68 70 71 M 72 73 74 75 M
Ava II sequence S S N N
mp result + + + + + S + S R R R R R + + + + + + + + + + +
A, uncut
B, cut
C, cut
D, cut
E, cut

## Slide 2
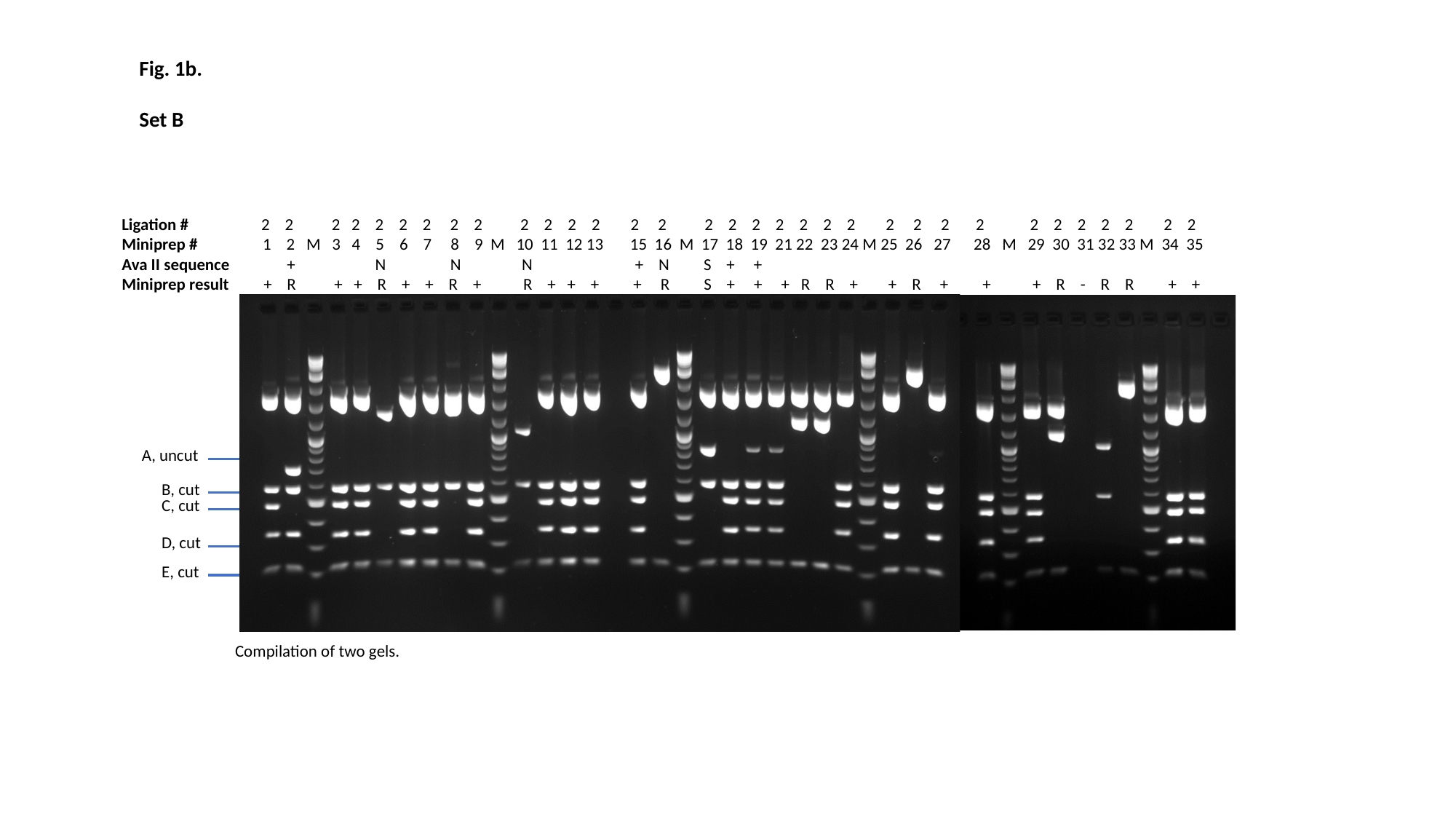

Fig. 1b.
Set B
Ligation # 2 2 2 2 2 2 2 2 2 2 2 2 2 2 2 2 2 2 2 2 2 2 2 2 2 2 2 2 2 2 2 2 2
Miniprep # 1 2 M 3 4 5 6 7 8 9 M 10 11 12 13 15 16 M 17 18 19 21 22 23 24 M 25 26 27 28 M 29 30 31 32 33 M 34 35
Ava II sequence + N N N + N S + +
Miniprep result + R + + R + + R + R + + + + R S + + + R R + + R + + + R - R R + +
A, uncut
B, cut
C, cut
D, cut
E, cut
Compilation of two gels.

## Slide 3
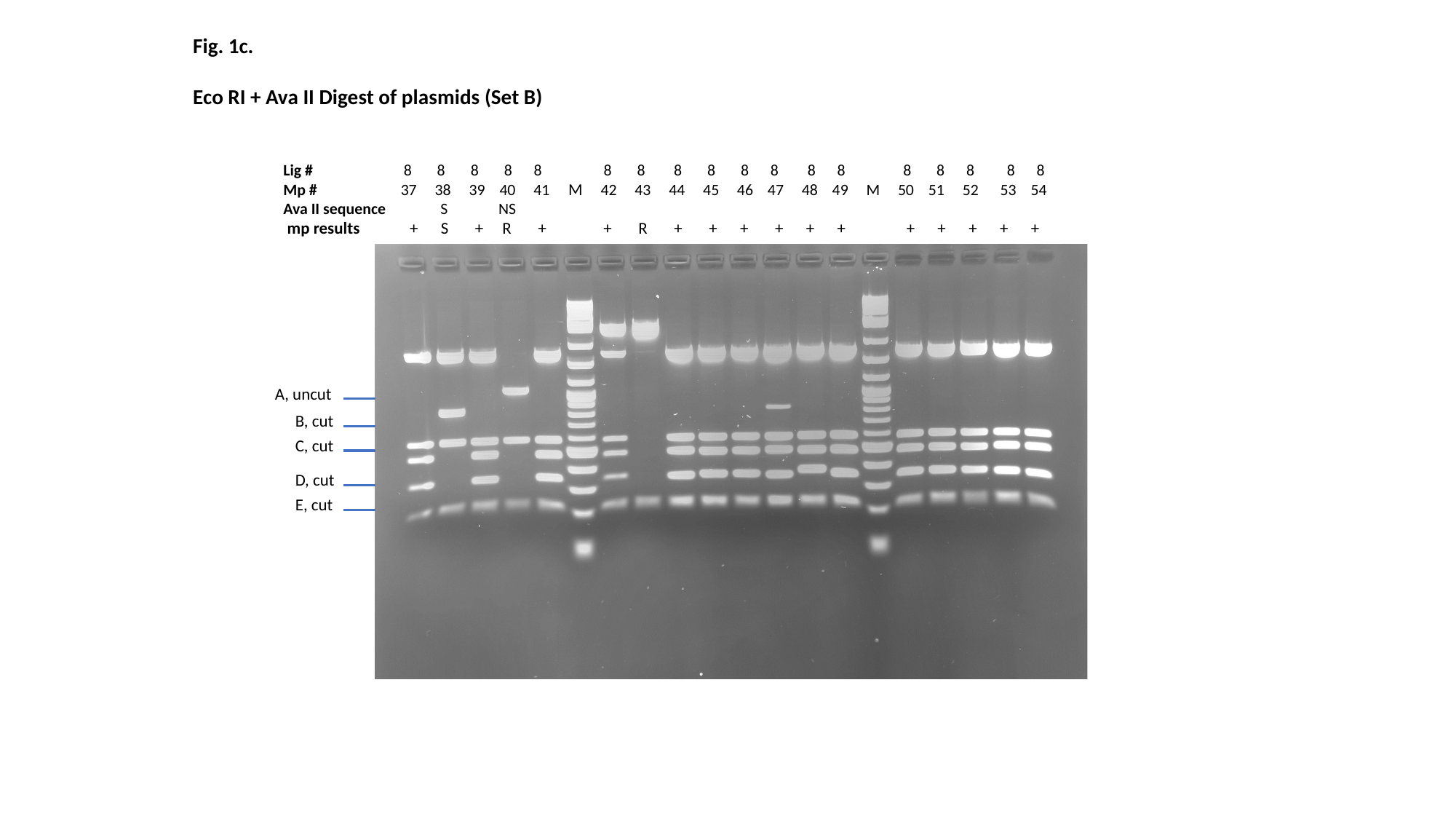

Fig. 1c.
Eco RI + Ava II Digest of plasmids (Set B)
Lig # 8 8 8 8 8 8 8 8 8 8 8 8 8 8 8 8 8 8
Mp # 37 38 39 40 41 M 42 43 44 45 46 47 48 49 M 50 51 52 53 54
Ava II sequence S NS
 mp results + S + R + + R + + + + + + + + + + +
A, uncut
B, cut
C, cut
D, cut
E, cut

## Slide 4
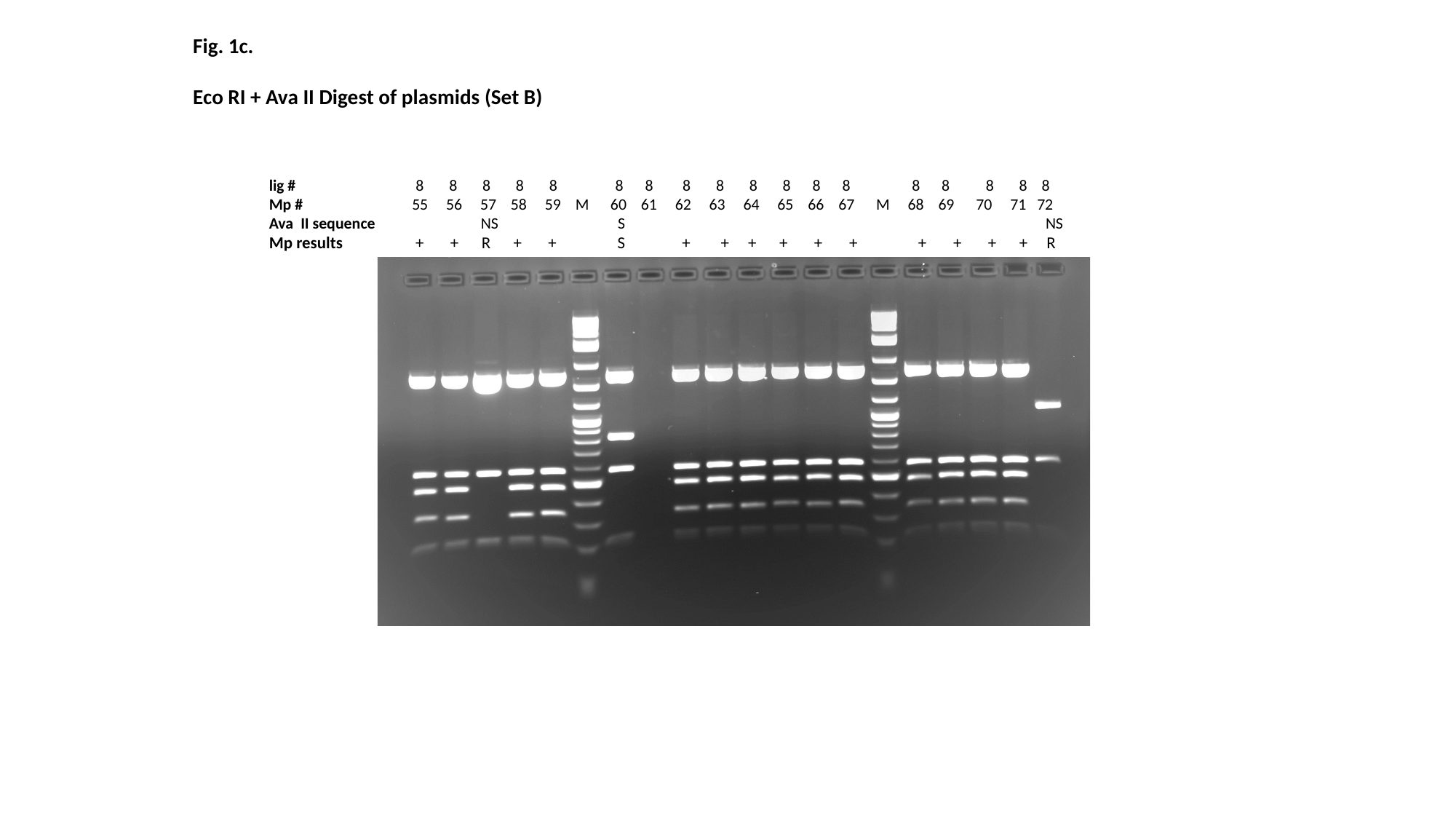

Fig. 1c.
Eco RI + Ava II Digest of plasmids (Set B)
lig # 8 8 8 8 8 8 8 8 8 8 8 8 8 8 8 8 8 8
Mp # 55 56 57 58 59 M 60 61 62 63 64 65 66 67 M 68 69 70 71 72
Ava II sequence NS S NS
Mp results + + R + + S + + + + + + + + + + R

## Slide 5
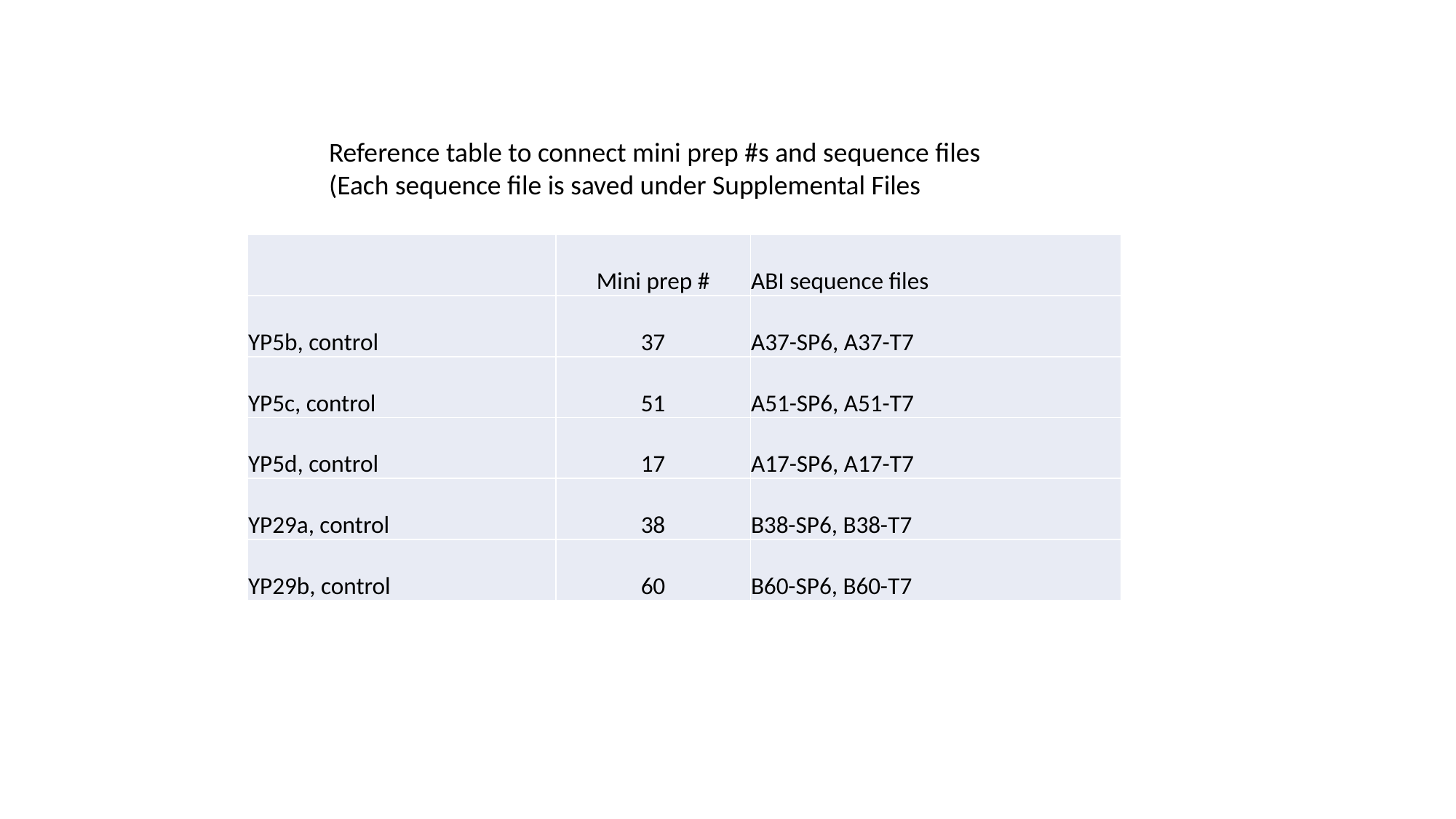

Reference table to connect mini prep #s and sequence files
(Each sequence file is saved under Supplemental Files
| | Mini prep # | ABI sequence files |
| --- | --- | --- |
| YP5b, control | 37 | A37-SP6, A37-T7 |
| YP5c, control | 51 | A51-SP6, A51-T7 |
| YP5d, control | 17 | A17-SP6, A17-T7 |
| YP29a, control | 38 | B38-SP6, B38-T7 |
| YP29b, control | 60 | B60-SP6, B60-T7 |
